# Supplementary material for: PROMPTa—A multifaceted assessment approach to develop writing skills
Source: PLoS One. 2025 Dec 2;20(12):e0337342. doi: 10.1371/journal.pone.0337342 (PMC12671783; doi:10.1371/journal.pone.0337342)
Supplement: Attachment — Submitted filename: PONE-D-25-23770_reviewer (1).pdf [file pone.0337342.s003.pdf]

PLOS ONE

PROMPTa - A Multifaceted Assessment Approach to Develop Writing Skills

--Manuscript Draft--

**Manuscript Number:**	PONE-D-25-23770
**Article Type:**	Research Article
**Full Title:**	PROMPTa - A Multifaceted Assessment Approach to Develop Writing Skills
**Short Title:**	PROMPTa - A Multifaceted Assessment Approach to Develop Writing Skills
**Corresponding Author:**	Noel Anurag Prashanth Nittala, PhD<br>IIIT DM Kurnool: Indian Institute of Information Technology Design and Manufacturing Kurnool<br>Kurnool, Andhra Pradesh INDIA
**Keywords:**	PROMPTa (Performance Review of Manuscript Proficiency Tool for Assessment); Alternative Assessment; Peer-assessment and feedback; Mentor Assessment and feedback; Expert assessment and feedback; Writing Skills; and ICT.
**Abstract:**	Assessment is essential for evaluating writing skills but traditional methods such as teacher grading alone are not enough. A more comprehensive approach is needed. This study explores the impact of alternative assessment methods such as peer, mentor, and expert assessment and feedback, on writing skill development in Nagaland. It also examines the role of ICT integration through PROMPTa (Performance Review of Manuscript Proficiency Tool for Assessment) in enhancing writing proficiency. The sample consisted of 143 first-year undergraduates, with 92 selected based on standard deviation of the mean forming a homogeneous sample. Pre- test scores (M = 13.28 vs. M = 13.41) confirmed homogeneity. Students were randomly divided into an experimental group (n=46), which used PROMPTa for assessment, and a control group (n=46), which followed traditional classroom evaluation. The experimental group showed significant improvement, with higher post-test scores (M = 16.91) than the control group (M = 13.65), showing a mean difference of 3.26. Statistical analyses further validated the findings, intra-rater reliability (r = 0.667–0.743, p < 0.001) and inter-rater reliability (r = 0.569–0.752, p < 0.001). These results highlight the effectiveness of ICT-based alternative assessments in enhancing writing skills.
**Order of Authors:**	Vezolu Puro
	Noel Anurag Prashanth Nittala, PhD
	Hariharasudan A
**Additional Information:**	
**Question**	**Response**
**Financial Disclosure**<br><br>Enter a financial disclosure statement that describes the sources of funding for the work included in this submission. Review the submission guidelines for detailed requirements. View published research articles from *PLOS ONE* for specific examples.<br><br>This statement is required for submission and **will appear in the published article** if the submission is accepted. Please make sure it is accurate.	The author(s) received no specific funding for this work.

Powered by Editorial Manager® and ProduXion Manager® from Aries Systems Corporation

**Funded studies**<br>Enter a statement with the following details:<br>• Initials of the authors who received each award<br>• Grant numbers awarded to each author<br>• The full name of each funder<br>• URL of each funder website<br>• Did the sponsors or funders play any role in the study design, data collection and analysis, decision to publish, or preparation of the manuscript?<br><br>Did you receive funding for this work?	
**Competing Interests**<br><br>Use the instructions below to enter a competing interest statement for this submission. On behalf of all authors, disclose any competing interests that could be perceived to bias this work—acknowledging all financial support and any other relevant financial or non-financial competing interests.<br><br>This statement is required for submission and **will appear in the published article** if the submission is accepted. Please make sure it is accurate and that any funding sources listed in your Funding Information later in the submission form are also declared in your Financial Disclosure statement.<br><br>View published research articles from *PLOS ONE* for specific examples.	The authors have declared that no competing interests exist.

Powered by Editorial Manager® and ProduXion Manager® from Aries Systems Corporation

**NO authors have competing interests**<br><br>Enter: *The authors have declared that no competing interests exist.*<br><br>**Authors with competing interests**<br><br>Enter competing interest details beginning with this statement:<br><br>*I have read the journal's policy and the authors of this manuscript have the following competing interests: [insert competing interests here]*<br><br>* typeset	
**Ethics Statement**<br><br>Enter an ethics statement for this submission. This statement is required if the study involved:<br><br>• Human participants<br>• Human specimens or tissue<br>• Vertebrate animals or cephalopods<br>• Vertebrate embryos or tissues<br>• Field research<br><br>Write "N/A" if the submission does not require an ethics statement.<br><br>General guidance is provided below. Consult the submission guidelines for detailed instructions. **Make sure that all information entered here is included in the Methods section of the manuscript.**	Human Subject Research:<br><br>The study was granted ethical clearance by the ethics committee of the Capital College of Higher Education with the approval number ECR/01/Inst/NG/2023. In addition, written consent was obtained from all the participants through a questionnaire during the study. The data collected were codified and analyzed anonymously.

Powered by Editorial Manager® and ProduXion Manager® from Aries Systems Corporation

**Format for specific study types**<br><br>**Human Subject Research (involving human participants and/or tissue)**<br>• Give the name of the institutional review board or ethics committee that approved the study<br>• Include the approval number and/or a statement indicating approval of this research<br>• Indicate the form of consent obtained (written/oral) or the reason that consent was not obtained (e.g. the data were analyzed anonymously)<br><br>**Animal Research (involving vertebrate animals, embryos or tissues)**<br>• Provide the name of the Institutional Animal Care and Use Committee (IACUC) or other relevant ethics board that reviewed the study protocol, and indicate whether they approved this research or granted a formal waiver of ethical approval<br>• Include an approval number if one was obtained<br>• If the study involved *non-human primates*, add *additional details* about animal welfare and steps taken to ameliorate suffering<br>• If anesthesia, euthanasia, or any kind of animal sacrifice is part of the study, include briefly which substances and/or methods were applied<br><br>**Field Research**<br><br>Include the following details if this study involves the collection of plant, animal, or other materials from a natural setting:<br>• Field permit number<br>• Name of the institution or relevant body that granted permission	
**Data Availability**<br><br>Authors are required to make all data underlying the findings described fully available, without restriction, and from the time of publication. PLOS allows rare exceptions to address legal and ethical concerns. See the PLOS Data Policy and FAQ for detailed information.	Yes - all data are fully available without restriction

A Data Availability Statement describing where the data can be found is required at submission. Your answers to this question constitute the Data Availability Statement and **will be published in the article**, if accepted.<br><br>**Important:** Stating 'data available on request from the author' is not sufficient. If your data are only available upon request, select 'No' for the first question and explain your exceptional situation in the text box.<br><br>Do the authors confirm that all data underlying the findings described in their manuscript are fully available without restriction?	
**Describe where the data may be found in full sentences. If you are copying our sample text, replace any instances of XXX with the appropriate details.**<br><br>• If the data are **held or will be held in a public repository**, include URLs, accession numbers or DOIs. If this information will only be available after acceptance, indicate this by ticking the box below. For example: *All XXX files are available from the XXX database (accession number(s) XXX, XXX.)*.<br>• If the data are all contained **within the manuscript and/or Supporting Information files**, enter the following: *All relevant data are within the manuscript and its Supporting Information files.*<br>• If neither of these applies but you are able to provide **details of access elsewhere**, with or without limitations, please do so. For example:<br><br>*Data cannot be shared publicly because of [XXX]. Data are available from the XXX Institutional Data Access / Ethics Committee (contact via XXX) for researchers who meet the criteria for access to confidential data.*<br><br>*The data underlying the results presented in the study are available from (include the name of the third party*	All relevant data are within the manuscript and its Supporting Information files.

and contact information or URL).<br>• This text is appropriate if the data are owned by a third party and authors do not have permission to share the data.<br><br>* typeset	
Additional data availability information:	

Manuscript Click here to access/download;Manuscript;Manuscript.docx

PROMPTa - A Multifaceted Assessment Approach to Develop Writing Skills

Vezolu Puro[1], Noel Anurag Prashanth Nittala[1], Hariharasudan.A[2]

[1]Department of Sciences, Indian Institute of Information Technology Design and Manufacturing, Jagannathagattu Hill Kurnool - 518008, Andhra Pradesh, India.

[2]Professor, School of Liberal Arts and Special Education (SLASE), Kalasalingam Academy of Research and Education, Kurishnankoil,Tamil Nadu, India.

***Corresponding Author's Email**: noel@iiitk.ac.in

Corresponding Author's Address

Dr. Noel Anurag Prashanth Nittala

Assistant Professor,

Department of Sciences - English,

Indian Institute of Information Technology Design and Manufacturing,

Jagannathagattu Hill, Kurnool - 518008,

Andhra Pradesh, India.

Email: noel@iiitk.ac.in

Mobile: +919700940242

ORCID IDs & Email IDs of all the Authors

Vezolu Puro, 322dso0001@iiitk.ac.in, ORCID:0009-0004-6985-6023

Noel Anurag Prashanth Nittala, noel@iiitk.ac.in, ORCID: 0000-0002-1889-911X

Hariharasudan, A., a.hariharasudan@klu.ac.in, ORCID: 0000-0002-7938-9383

Acknowledgments

The authors are thankful to the participants of the study and the management of the institution where the study was conducted.

Author Contributions

Vezolu Puro: Conceptualization, Formal Analysis, Investigation, Methodology, Validation and Writing-Original draft

Noel Anurag Prashanth Nittala: Conceptualization, Methodology, Supervision, Visualization and Writing-review & Editing.

Hariharasudan. A: Data Curation, Formal Analysis, Software, Validation and Writing-review & Editing.

Declarations

Consent to Participate

Permission was issued by the Institution where the data was collected, and ethical committee clearance was obtained. Written consent from the participants was also obtained. All the participants were adults.

Conflict of Interest:

The authors declared no potential conflicts of interest with respect to the research, authorship, and/or publication of this article.

Funding

The authors received no financial support for the research, authorship, and/or publication of this article.

Data availability

All data underlying the findings of the study described in the manuscript are fully available without restriction with the corresponding author.

Abstract

Assessment is essential for evaluating writing skills but traditional methods such as teacher grading alone are not enough. A more comprehensive approach is needed. This study explores the impact of alternative assessment methods such as peer, mentor, and expert assessment and feedback, on writing skill development in Nagaland. It also examines the role of ICT integration through PROMPTa (Performance Review of Manuscript Proficiency Tool for Assessment) in enhancing writing proficiency. The sample consisted of 143 first-year undergraduates, with 92 selected based on standard deviation of the mean forming a homogeneous sample. Pre- test scores ($M = 13.28$ vs. $M = 13.41$) confirmed homogeneity. Students were randomly divided into an experimental group ($n=46$), which used PROMPTa for assessment, and a control group ($n=46$), which followed traditional classroom evaluation. The experimental group showed significant improvement, with higher post-test scores ($M = 16.91$) than the control group ($M = 13.65$), showing a mean difference of 3.26. Statistical analyses further validated the findings, intra-rater reliability ($r = 0.667$–0.743, $p < 0.001$) and inter-rater reliability ($r = 0.569$–0.752, $p < 0.001$). These results highlight the effectiveness of ICT-based alternative assessments in enhancing writing skills.

Keywords: PROMPTa (Performance Review of Manuscript Proficiency Tool for Assessment)**,** Alternative Assessment, Peer-assessment and feedback, Mentor Assessment and feedback, Expert assessment and feedback, Writing Skills, and ICT.

Abbreviations:

AECC: Ability Enhancement Compulsory Course

CBCS: Choice-Based Credit System

FYUGP: Four-Year Undergraduate Program

ICT: Information and Communication Technology

NCF: National Curriculum Framework

NEP: National Education Policy

PROMPTa: Performance Review of Manuscript Proficiency Tool for Assessment

SCT: Social Cognitive Theory

SPSS: Statistical Package for the Social Sciences

1. **Introduction**

Effective communication is built on a foundation of essential skills, with writing [1] being one of the most crucial skills for academic success and professional development. According to Hyland [2], writing constructs a coherent social reality by enabling interaction. Among the four language skills: listening, speaking, reading, and writing, writing skill stands out as the most complex [3]. Unlike other skills, it requires a mastery of both linguistic competency over grammar and vocabulary, and communicative competence- the ability to convey ideas clearly and appropriately for a specific audience [4]. Writing also involves the integration of intellectual abilities, cognitive strategies, language rules, and motivation [5 & 6]. This makes it a multidimensional skill where learners must adhere to language rules, structure information logically, and transform knowledge into clear, meaningful communication [7].

Given these cognitive demands, writing serves as a modality for assessing academic achievement, especially in higher education [8]. Through writing, students demonstrate intellectual development, critical thinking, and the ability to articulate complex ideas. Unlike assessments that focus solely on content recall, writing evaluates a student's ability to

synthesize and organize knowledge into coherent arguments, making it an indispensable tool for measuring academic achievement and intellectual growth. Despite its importance, developing proficient writing skills remains a challenge for both students and teachers alike [9,3]. This challenge is particularly evident in Nagaland, where, despite having a high literacy rate, the educational system struggles to equip students with effective writing skills. Even though the reasons for such are varied, the traditional approach to learning can be seen as a contributing factor.

To address these challenges, a more multifaceted approach to writing assessment is needed—one that integrates peer assessment, mentor feedback, and expert reviews to provide a holistic and constructive evaluation. This approach, facilitated by Information and Communication Technologies (ICT), offers opportunities for iterative learning, allowing students to receive diverse perspectives and engage in a continuous cycle of reflection, revision, and improvement. Traditional summative assessments, which focus on content recall, do not fully capture the learner's ability to think and engage critically to produce meaningful and well-organized written work. Instead of evaluating the final product, it is important to focus on the process of learning itself. This is where formative assessment becomes essential as it not only evaluates the writing development but also emphasizes the writing process, where ongoing feedback and guidance are given to support learners' growth.

The digital age has transformed writing production and assessment through ICT integration. Digital platforms expand writing beyond traditional writing conventions, making adaptability an essential skill. While digital tools enhance efficiency, they also raise concerns about originality and academic integrity. Artificial Intelligence (AI) further transformed writing, introducing new challenges. Increasing reliance on AI raises questions and difficulties for educators to determine whether a learner's work reflects genuine effort, critical thinking and intellectual engagement As a result, writing assessments must emphasize higher-order

skills such as creativity, critical thinking and originality which are uniquely human cognitive abilities and not replicated by AI, ensuring learners develop analytical and expressive skills for effective communication.

As writing evolves in the digital age, assessment practices must also adapt. This study addresses these challenges by exploring the role of peer, mentor, and expert assessment in improving undergraduate writing skills in Nagaland. Through the integration of ICT-driven assessments, it aims to highlight how different feedback mechanisms support student writing development. Hence, this study explores the effectiveness of PROMPTa (Performance Review of Manuscript Proficiency Tool for Assessment), an ICT-based assessment tool designed to facilitate feedback from peers, mentors, and experts. PROMPTa enables learners to receive and give detailed, scaffolded feedback at different stages of the writing process, making assessments more interactive and personalized. Through its integration, the study aims to assess its impact on writing proficiency.

1.1 Background of the Study

Nagaland, a state in Northeast India, presents a unique education system because of its multilingual composition, characterized by its multi-tribal inhabitants, each with distinct languages and traditions. This multilingual setting poses significant challenges for education. While English has emerged as the primary medium of instruction, its effective integration within the educational framework remains a challenge. Nagamese, a local pidgin, often serves as the medium for classroom interactions, creating a gap between what is officially taught and what is spoken. Despite an emphasis on English learning, research [10] highlights that many learners still struggle with the language. One reason is the treatment of English as a subject rather than a skill [11], leading to low proficiency in the language, particularly in writing, as they struggle to improve their writing and express their thoughts effectively.

Nagaland's education system contrasts a high literacy rate and a deeply rooted traditional approach. Described as a "one-way traffic" [12] system, education in Nagaland is teacher-centered, leaving learners as passive recipients of information "the present system of education has failed to empower learners" [10]. This approach, reflective of Gardner's "Uniform view of Schooling" [13] disregards individual learning needs and emphasizes conformity and standardized methods. This reinforces rote learning and memorization, limiting opportunities for creativity and independent thought. It creates a learning environment where students become proficient at reproducing information but struggle with independent thought and expression. A particularly concerning aspect of the current system is the approach to writing instruction, as teachers emphasize grammar, structure, and correctness over content and expression. This misplaced emphasis is especially evident at the higher secondary level [14], where students continue to rely heavily on teacher explanations and textual interpretations. Writing is often limited to taking down dictated notes, with little opportunity for independent thought or expression. Writing is viewed primarily as a way to achieve grades, rather than a tool for communication and creativity [15-17]. A lack of confidence in students' writing abilities leads teachers to rely on dictation [18], reinforcing passive learning and disengagement. Consequently, writing becomes disconnected from real-life contexts, with many students perceiving it as difficult and uninteresting [19].

The assessment practices in Nagaland worsens these challenges. The predominant use of traditional summative assessments, with their focus on memorization and standardized responses, fails to capture the iterative nature of the writing process, including crucial elements such as drafting, reflection, and revision [20]. This approach neglects the development of higher-order thinking skills—analysis, synthesis, and evaluation—essential for proficient writing. Motivation, a key predictor of writing performance [21 & 22], is also hindered by the rigid pedagogy. However, the prevailing pedagogy, characterized by limited feedback practices

and focus on error corrections rather than meaningful interaction and constructive guidance, leaves students struggling to develop the confidence and skills needed for effective writing, creating a culture of anxiety and fear around writing tasks [23].

The necessity for change aligns with national educational priorities, as outlined in both the National Curriculum Framework (NCF) 2005 and the National Education Policy (NEP) 2020. These emphasize formative assessment as a key tool for learning and skill development. The NEP 2020 advocates for a shift from summative to formative approaches, promoting continuous evaluation to support holistic learning. Integrating ICT tools and alternative assessment methods can address Nagaland's specific educational challenges while aligning with modern demands. This study explores the transition from traditional summative assessments to formative, learner-centered approaches, including peer assessment and feedback, mentor assessment and feedback, and expert assessment and feedback.

1.2 Theoretical Framework

The study builds upon the theoretical foundations of Social Cognitive Theory (SCT), the Social Cognitive Model of Writing, Constructivism, and Constructionism to investigate how alternative assessment methods, such as peer assessment and feedback, mentor assessment and feedback, and expert assessment and feedback, enhance writing skills through ICT-based intervention. SCT, as proposed by Bandura [24] highlights the centrality of self-efficacy in influencing human motivation, emotional regulation and behaviour with its development influenced by four primary sources: (a) performance accomplishments (i.e., successful accomplishments raise mastery expectations while repeated failures lower these), (b) vicarious experience (i.e., observing identifiable models who succeed or fail), (c) verbal persuasion (i.e., being encouraged to believe in oneself), and (d) emotional arousal (i.e., relating stressful situations to personal performance). This theory underscores the importance of social and environmental roles in influencing an individual's belief in their ability to perform tasks, which

is particularly relevant to writing, where feedback and collaborative learning are essential. Building on SCT, Zimmerman and Risemberg [25] recognize writing as a dynamic, self-regulated process that integrates personal, behavioural, and environmental strategies. This is done through multiple rounds of feedback. On receiving feedback, writers review their work and refine their work through necessary changes. Cognitive writing strategies, such as structuring ideas, revising work, which are essential for improving writing skills, can be strengthened through explicit instruction and learning from peers [24 & 25].

Supporting the above, Constructivism [26 & 27] explains that learning happens best when new knowledge connects with past experiences in a meaningful way. Instead of simply memorising information, learners actively build their understanding through experimentation, questioning, and application of their knowledge, encouraging independent learning and critical thinking. Peer assessment promotes such learning as it allows learners to review each other's work and exchange ideas, refining their understanding through interaction and reflection, and developing deeper insights through different perspectives. Constructionism [28] builds on this idea by emphasizing "learning by making," where learners learn through hands-on, creative activities. This is relevant in writing as learners improve their writing skills through actively engaging in an interactive process of applying their creativity, receiving and giving meaningful feedback, and refining their work through multiple drafts. When these approaches are applied in a technology-supported environment, learning becomes more interactive and effective. ICT tools make it easier for learners to collaborate, share feedback, and monitor their learning progress.

1.3 Literature Review: Alternative Assessment in Improving Writing Skills through ICT`

To improve writing skills, assessment should not only focus on the final product but also on the writing process and progress. In a language classroom, assessment should help learners demonstrate what they have learned rather than what they have not learned. In this

vein, alternative assessment focuses on "assessment for learning," which helps learners understand how they learn and apply their knowledge [29].

Alternative assessment encourages active participation, reflection, and continuous improvement, making it particularly effective in teaching writing. Approaches such as peer, mentor, and expert feedback facilitate an interactive process where learners can revise and refine their work based on constructive input. Some effective methods include self-assessment [30 & 31], portfolio assessments [32 & 33], and teacher-student conferences [34 & 35].

Research shows that peer assessment is highly effective for improving writing skills as it allows learners to collaborate in planning, revising, and composing texts, making learning more interactive and reflective [36]. It also promotes a process approach to writing as it facilitates negotiation, decision making, communication, and critical thinking [37- 42]. A study by Tinh [3] found that learners who regularly used peer review checklists improved their writing skills significantly. However, Peer assessment has its own challenges. Studies suggest that its effectiveness depends on factors like the number of peers involved, low quality of feedback, and trust among the peers [43 & 44]. There are also concerns about bias and reliability [45-47].

The introduction of ICT has expanded peer assessment, making it more flexible and accessible. Online peer feedback has demonstrated improvements in learning outcomes [48-52]. ICT-supported peer feedback provides a comfortable learning environment, encouraging self-regulation and self-direction [53 & 54]. It also enhances critical thinking [55] and increases peer commentary, leading to more effective revisions [56]. One of the primary reasons for its success is its motivational impact, reducing learner pressure and enabling freer expression [57 & 58]. Furthermore, ICT minimizes time and space limitations, facilitating peer feedback [59]. Despite these advantages, ICT-facilitated peer feedback presents challenges. Anonymity can sometimes hinder the specificity of feedback [59]. Learners may also struggle with trust in peer

evaluations, resistance to critical feedback, and low feedback literacy [60-62]. Providing constructive peer feedback requires higher-order thinking skills, which some students may lack [63 & 64].

A structured peer assessment process enhances its effectiveness. Clear assessment criteria help students understand what to focus on when reviewing peers' work. Training on constructive feedback and instructor oversight ensures that feedback remains fair and useful. A structured approach encourages an interactive and supportive learning environment, helping learners improve writing skills while developing analytical abilities. Peer assessment promotes responsibility for learning, aligning with modern pedagogies that emphasize independence and active participation. It cultivates critical thinking, communication, and self-reflection skills, making learners more engaged and effective. Writing is more than an individual task; it is a social activity that involves communication between the writer and the instructor [65]. Alongside peer assessment and feedback, teacher feedback, be it oral [46, 66, 65] or written [67 & 68] as well as expert assessment and feedback [69-72] play significant role in developing writing skills.

As part of educational reforms, new policies such as the National Curriculum Framework 2005 (NCF) and National Education Policy 2020 (NEP) have been introduced to improve student learning. These policies emphasise a shift from traditional to formative assessment approaches, focusing on how learners learn rather than solely grading them. In this process, alternative assessment methods are encouraged as they provide a more comprehensive and holistic assessment of the learner. With Nagaland adopting policies like the Four-Year Undergraduate Program (FYUGP) and Choice-Based Credit System (CBCS), there is a transition from rote memorization to skill-based learning. The NEP 2020 further advocates for integrating technology in education, promoting competency-based assessments. Despite these reforms, Nagaland faces many challenges, particularly ICT integration into the classrooms,

including inadequate infrastructure, limited connectivity, accessibility barriers, and insufficient training for teachers as well as students. Although initiatives like the e-Naga Summit (2015) promoted ICT adoption, gaps between policies and practice remain. The Nagaland Sustainable Development Goal Vision 2030 has emphasized the need for better teacher training and improved access to quality education. In such light, this study explores the potential of alternative assessment—peer, mentor, and expert assessment and feedback—facilitated by ICT. This paper, thus, situates its need and significance within this evolving educational context, advocating for a well-structured, technology-supported approach to alternative assessment in writing instruction.

1.4 Study Objectives

1. To investigate the impact of alternative assessment methods, such as peer assessment and feedback, mentor assessment and feedback, and expert assessment and feedback, on the development of writing skills in the context of Nagaland.

2. To assess the impact of ICT integration in supporting writing skill enhancement through the implementation of alternative assessment methods.

1.5 Null Hypotheses

1. There is no significant difference in the improvement of writing skills between learners who receive alternative assessments and those who receive traditional assessment methods.

2. The use of ICT-integrated alternative assessments does not significantly improve writing outcomes compared to traditional approaches without ICT.

2. Methodology

The study used a mixed-methods experimental design with a control and an experimental group to examine the effectiveness of alternative assessment methods in developing writing skills. Both groups underwent pre-test and post-test evaluations to measure progress. The experimental group received an eight-week intervention between 17-07-2023 to 09-09-2023, through the online platform PROMPTa as part of their Ability Enhancement Compulsory Course (AECC). The first week included orientation, a pre-test, a questionnaire, and an introduction to writing concepts to both groups. In the second week, the experimental group was introduced to alternative assessment methods using PROMPTa, supported by brainstorming activities, while the control group followed the traditional approach. Weeks 3 to 7 focused on three writing tasks, namely, communicative, creative, and academic, each involving multiple drafts and iterative revisions. The experimental group received assessments guided by rubrics as well as structured feedback from peers, mentors, and experts, with peer assessments conducted anonymously. The final week concluded with a post-test and a reflection session.

Both groups completed the same tasks, but their assessment methods differed. The control group followed traditional assessment practices, where the mentor assessed all drafts. The experimental group underwent a three-stage alternative assessment process, including peer assessment and feedback for critical thinking and self-reflection, mentor assessment and feedback for guidance, and expert assessment and feedback for higher-level refinement. While the control group's assessments were conducted offline, the experimental group used PROMPTa for an entirely digital assessment process. A post-test at the end of the eight weeks measured writing proficiency improvements. Qualitative data were also collected through

follow-up questionnaires and semi-structured interviews to assess perceptions and experiences of assessment methods and evaluate PROMPTa's effectiveness.

A multi-layered feedback approach was implemented to address the limitations of peer feedback and help learners improve their writing step by step. First, peer assessment and feedback were aimed at helping learners identify general areas for improvement. This was followed by mentor assessment and feedback to provide a more focused and personalised feedback. Finally, experts assessed the work and offered professional insights to refine their work further. By receiving continuous feedback at different levels, the study aimed to help learners understand the writing process and gradually improve their writing skills.

2.1 Participants of the Study

The selection of the sample was based on their need for foundational writing skills development. A total of 143 first-year undergraduate students were initially selected from a College in Kohima, Nagaland. All participants completed a written composition pre-test to establish a baseline level of writing proficiency. To create a more homogeneous sample for the study, 92 students whose scores fell within one standard deviation of the mean were selected and randomly assigned to experimental (online intervention) and control (Offline, traditional instruction) groups of equal size (n=46). Participants were informed about their involvement, and written consent was obtained through learner's questionnaire. All the participants were adults.

2.2 Tools

To ensure data triangulation, the study utilized a combination of questionnaires, interviews, writing tasks, and a web application called PROMPTa, developed specifically for the study. Questionnaires and semi-structured interviews were conducted to gather

perspectives on teaching instructions and assessment. Classroom observations provided insights into the existing classroom practices. In line with the focus of the study, writing tasks were used to understand the prevailing assessment practices, facilitate comparisons between traditional teaching and assessment practices over the proposed alternative assessment approach through peer, mentor, and expert feedback, and facilitate a comparison between traditional and ICT-based teaching methods. The writing tasks encompassed communicative, creative, and academic writing styles, including prompts and supplementary materials for reference and content building. Rubrics were used for evaluation, while feedback forms were used to gather insights. The assessment focused on four main areas: Content, Structure and Organization, Language and Vocabulary, and Overall Impact. Each area was worth 10 marks, adding up to a total of 40 marks.

2.3 PROMPTa (Performance Review Of Manuscript Proficiency Tool for Assessment)

PROMPTa, a web application designed specifically for the study, serves as the primary digital platform for evaluation and feedback. PROMPTa differs from traditional approaches by prioritizing the cyclical nature of writing over solely evaluating the final product. The platform acknowledges that effective writing is a developmental process that requires ongoing refinement through revisions and redrafting. This aligns with the study's objectives, which explore how alternative assessment methods can encourage a better understanding of the writing process itself. The use of PROMPTa is important for the study as it provides a platform for alternative assessment as well as allows the exploration of how integrating ICT tools can enhance writing skills.

2.4 Implementation of PROMPTa in the study

Please check the position of the subtitles in relation to the content

In this study, PROMPTa serves as a structured submission platform for multi-tiered assessment. It relies on human-assessed feedback rather than automated feedback. Feedback is exchanged anonymously, and evaluation is done using analytical rubrics developed by language experts to ensure accuracy and consistency. These rubrics served as standardized guidelines, outlining clear criteria for assessing various aspects of the written drafts while allowing room for open-ended feedback to address individual strengths and areas for improvement.

The assessment process involved three writing tasks, each emphasizing a different writing style: email writing for communicative writing, story writing for creative writing, and essay writing for academic writing. These writing tasks together represent essential competencies required by learners in higher education and professional development. Table 1 below presents an overview of these tasks.

Table 1: Overview of the Tasks

Tasks	Draft 1	Draft 2	Draft 3
**Task 1**-Communicative Writing (Email)	Peer Assessment and Feedback	Mentor Assessment and Feedback	Expert Assessment and Feedback
**Task 2**- Creative Writing (Story)	Peer Assessment and Feedback	Mentor Assessment and Feedback	Expert Assessment and Feedback
**Task 3**-Academic Writing (Essay)	Peer Assessment and Feedback	Mentor Assessment and Feedback	Expert Assessment and Feedback

Each task included three different topics, each requiring learners to submit a total of nine written drafts. Each draft underwent three rounds of assessment: peer assessment and feedback, mentor assessment and feedback, and expert assessment and feedback. This iterative process allowed for the refinement of the written drafts.

3. Data Analysis and Discussion

Please check the position of the subtitles in relation to the content

The data was analysed through quantitative and qualitative methods. Quantitative data, as seen in Figure 1, were analyzed using SPSS software, starting with pre-test data to establish baseline comparisons and confirm the validity of group comparisons. Levene's test was conducted to check the homogeneity of variances between the groups. The reliability assessment utilized Pearson's correlation coefficient to examine both intra-rater reliability (consistency of individual ratings over time) and inter-rater reliability (agreement between different raters). Statistical significance testing was done to validate the observed correlations. To evaluate the intervention's effectiveness, t-tests were performed on post-test data to compare mean differences between groups. The quantitative analysis provided measurable outcomes of the intervention's impact.

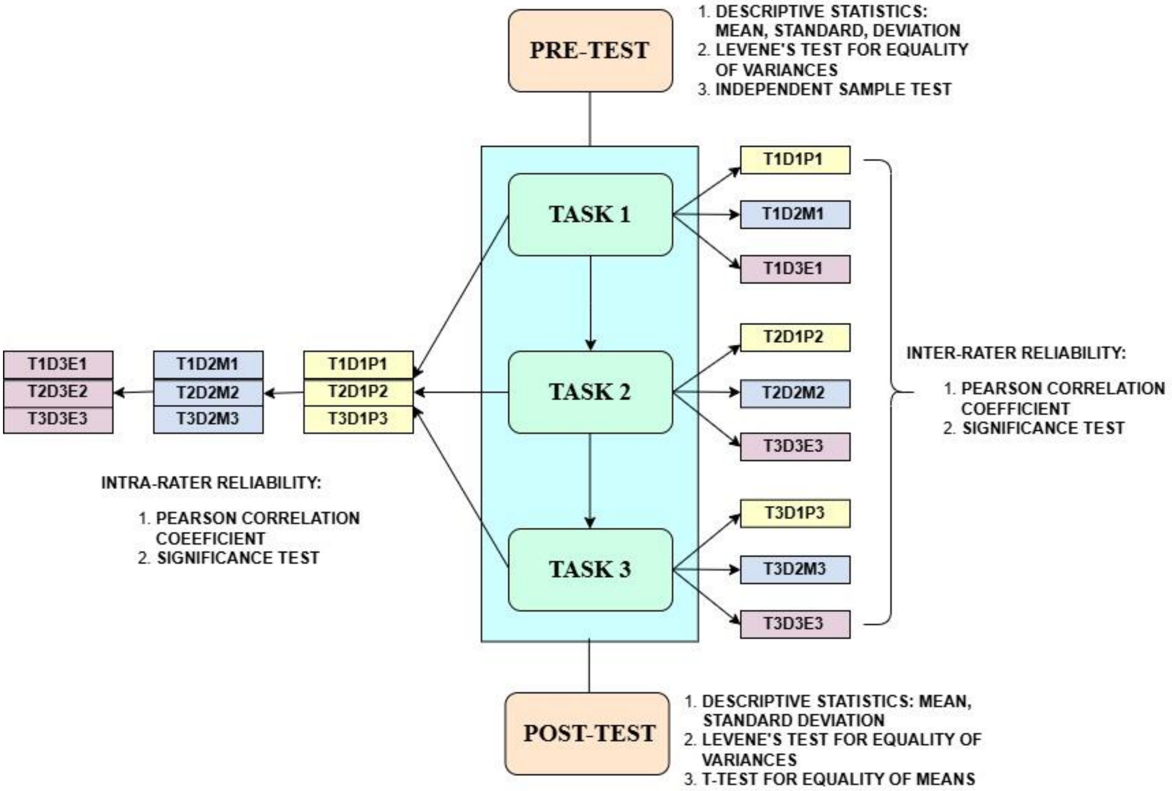

Figure 1: Quantitative Analysis
Note: T= Tasks, D=Drafts, P=Peer-assessment, M=Mentor Assessment and Feedback, E=Expert Assessment and Feedback

Complementing the statistical analysis, qualitative analysis was carried out through thematic analysis to identify patterns and insights. This dual analytical approach enabled a

comprehensive understanding of both the measurable outcomes and the contextual factors influencing the intervention's effectiveness. Alongside each analysis, the discussions of the findings are also presented in this section.

3.1 Quantitative Analysis

3.1.1 Results of Pre-Test

The pre-test was conducted to assess the initial proficiency levels of the learners and to establish homogeneity between the Experimental and Control groups before the intervention. The analysis included statistical tests to compare the groups' performance, and the results are shown in Table 2 and Figure 2.

Table 2: Pre-Test Results for Control Group and Experimental Group

Group	N	Mean	SD	Levene's Test for Equality of Variances		t	df	Sig.
Experimental	46	13.28	1.52	0.57	0.84	0.39	89	0.69
Control	46	13.41	1.45					

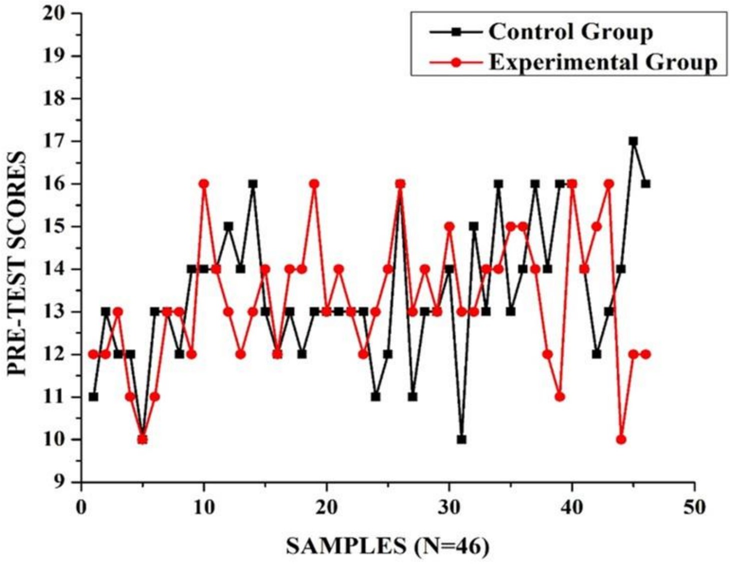

Figure 2: Graph for Control Group and Experimental Group (Pre-Test)

Levene's Test was done to determine if the variances between the two groups were equal. The results are as follows:

- p=0.57 (greater than 0.05), indicating no significant difference in variances.
- This confirms that the assumption of equal variances is met.

An independent samples t-test was also conducted to determine if there was a statistically significant difference in the mean scores between the Experimental and Control groups. The results are as follows:

- t=0.84, df= 89, p=0.69.
- The p-value is greater than 0.05, indicating no statistically significant difference in the means.

The pre-test results demonstrate that there were no significant differences in the proficiency of the Experimental group (M=13.28) and the Control group (M=13.41). The equality of variances further confirms the groups' homogeneity. This indicates that the two groups were comparable in their initial proficiency levels, providing a solid baseline for evaluating the effects of the intervention. By establishing homogeneity, the analysis ensures that any differences observed in the post-test can be attributed to the intervention rather than pre-existing differences between the groups. This baseline assessment is critical for the validity of subsequent comparisons.

3.1.2 Results of Reliability Estimates

To assess the consistency of post-test scores, both internal (intra) and external (inter) reliability measures were employed. This involved calculating the correlation between scores

assigned by the same rater for the same tasks. The correlation between scores provided by different raters for the different tasks were also calculated.

The following sections analyze the agreement and consistency among raters involved in an evaluation process. The data is presented in two sections: Intra-Rater Reliability and Inter-Rater Reliability.

3.1.3 Intra Reliability Estimates

Intra-rater reliability measures the consistency of scores assigned by the same rater across multiple evaluations of the same tasks. Table 3 and Figure 3 presents the Pearson correlation coefficients for the three rounds of ratings conducted by a single rater. The significance of these correlations is also reported.

Table 3: Estimates for Intra-Rater Reliability

Ratings	Correlation	First rating	Second rating	Third rating
First rating	Pearson correlation	1	0.743***	0.667***
	Sig. (2-tailed)	-	0.000	0.000
	N	46	46	46
Second rating	Pearson correlation	0.743***	1	0.693***
	Sig. (2-tailed)	0.000	-	0.000
	N	46	46	46
Third rating	Pearson correlation	0.667***	0.693***	1
	Sig. (2-tailed)	0.000	0.000	-
	N	46	46	46

**Correlation is significant at the 0.001 level (2-tailed).

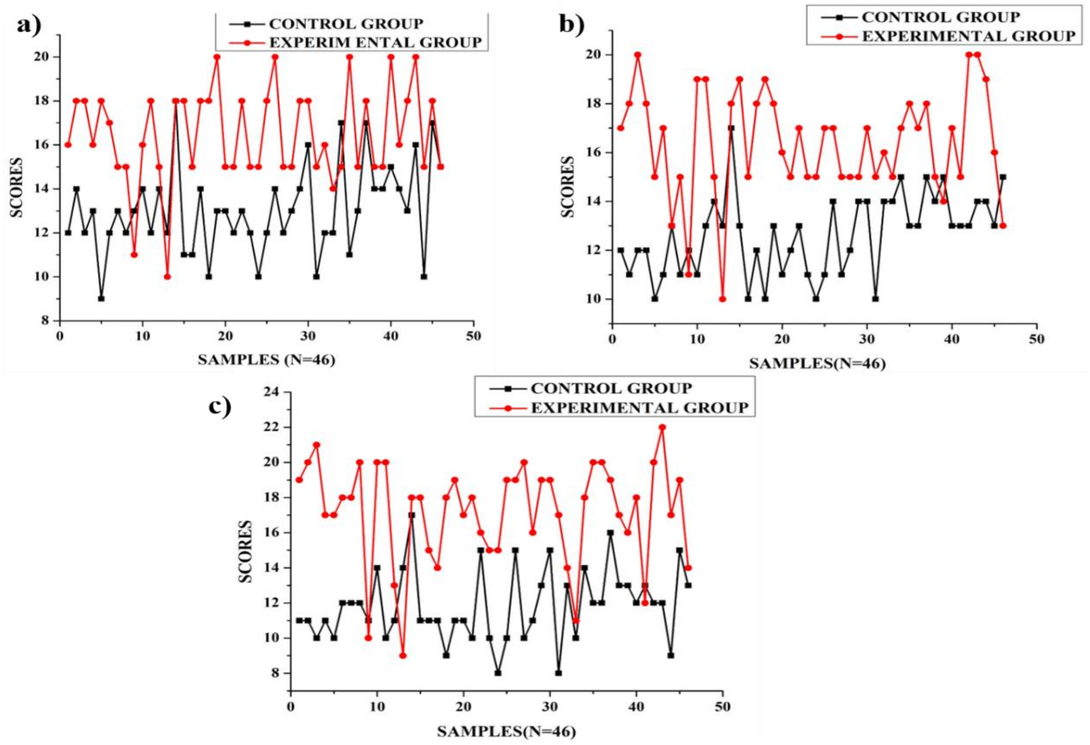

Figure 3: Intra-rater reliability estimates for control group and experimental group: (a) T1D2=Control Group, T1D2M1=Experimental Group, (b) T2D2=Control Group, T2D2M2= Experimental Group and (c) T3D2=Control Group, T3D2M3=Experimental Group

The intra-rater reliability analysis revealed strong positive correlations across all three rating rounds, indicating substantial consistency in the rater's evaluations over time. The strongest correlation was observed between the first and second ratings ($r = 0.743$, $p < 0.001$), followed by the second and third ratings ($r = 0.693$, $p < 0.001$), while the first and third ratings showed a slightly lower but still strong correlation ($r = 0.667$, $p < 0.001$). All correlations were statistically significant at the $p < 0.001$ level, demonstrating the relationships were not due to chance. The gradually decreasing correlation coefficients suggest a slight drift in scoring patterns over time, but the consistently strong correlations above 0.65 across all three rounds indicate the rater maintained reliable judgment throughout the evaluation process, lending credibility to the assessment outcomes. These results support the reliability of the intra-rater assessment process, ensuring the evaluator maintained a consistent standard while assigning scores.

3.1.4 Inter-Reliability Estimates

Inter-rater reliability measures the level of agreement and consistency among different raters evaluating the same tasks. Table 4 presents the Pearson correlation coefficients for ratings assigned by three different raters (Rater A, Rater B, and Rater C). The significance levels for the correlations are also reported.

Table 4**:** Estimates for Inter-Rater Reliability

**Raters**	**Correlation**	**Rater A**	**Rater B**	**Rater C**
Rater A	Pearson correlation	1	0.698***	0.569***
	Sig. (2-tailed)	-	0.000	0.000
	N	46	46	46
Rater B	Pearson correlation	0.698***	1	0.752***
	Sig. (2-tailed)	0.000	-	0.000
	N	46	46	46
Rater C	Pearson correlation	0.569***	0.752***	1
	Sig. (2-tailed)	0.000	0.000	-
	N	46	46	46

**Correlation is significant at the 0.001 level (2-tailed).

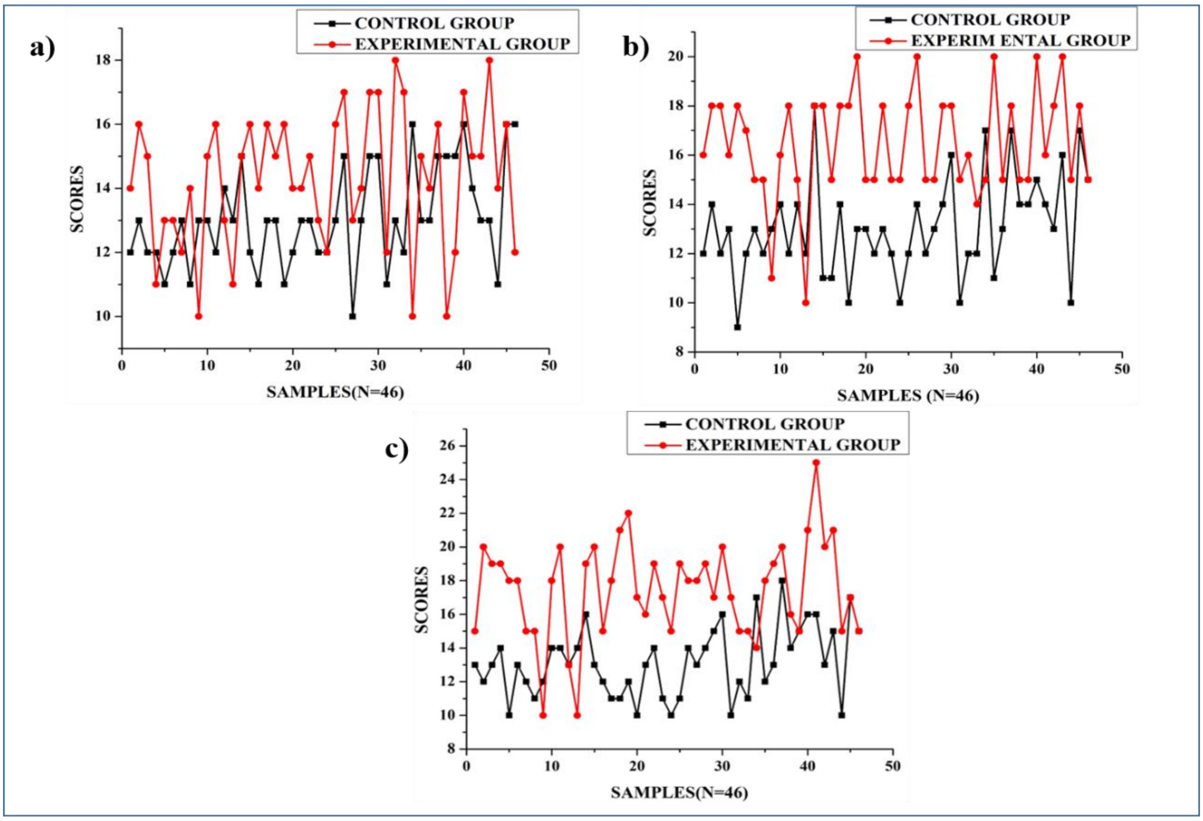

Figure 4: Inter-reliability for both control and experimental group, (a) T1D1=Control Group, T1D1P1=Experimental Group, (b) T1D2=Control Group, T1D2M1= Experimental Group, and (c) T1D3=Control Group, T1D3E1=Experimental Group

The inter-rater reliability analysis presented in Table 4 and Figure 4 indicates a moderate to strong level of agreement among the three raters (Rater A, Rater B, and Rater C), with correlation values ranging from 0.569 to 0.752. The highest agreement was observed between Rater B and Rater C (0.752), while the lowest, though still moderate, was between Rater A and Rater C (0.569). All correlations are statistically significant at the 0.001 level, confirming the reliability of the results and ensuring that the observed agreement is not due to chance.

3.1.5 Results of Post-Test

Table 5: Post-Test Results for Control Group and Experimental Group

Group	N	Mean	SD	Levene's Test for Equality of Variances		t	Df	Sig.	Mean Difference
Experimental	46	16.91	3.02	7.11	0.009	5.88	81	0.000	3.26
Control	46	13.65	2.15						

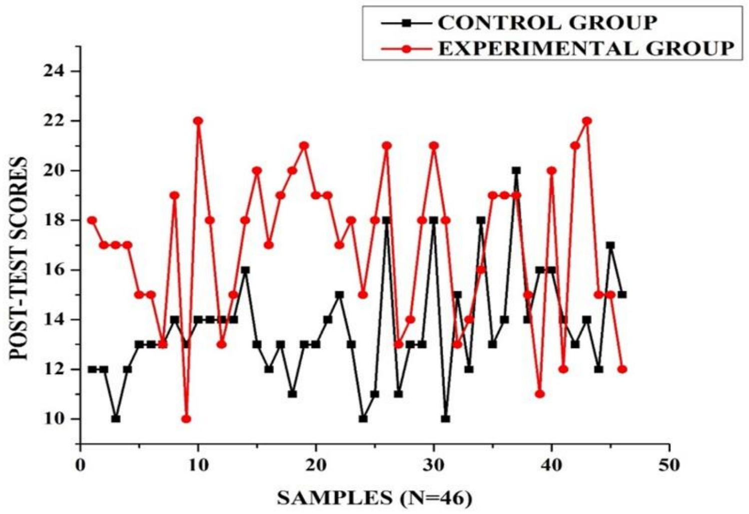

Figure 5: Graph for Control Group and Experimental Group (Post-Test)

The results in Table 5 and Figure 5 show a significant difference between the experimental (M = 16.91, SD = 3.02) and control groups (M = 13.65, SD = 2.15) in post-test scores, with the experimental group outperforming the control group. The mean difference of 3.26 points was statistically significant ($t(81) = 5.88$, $p < .001$), indicating the intervention's effectiveness. Greater variability in the experimental group's scores (SD = 3.02) suggests differing impacts among participants. Levene's test confirmed unequal variance ($p = 0.009$), reflecting diverse response patterns. Despite this, the experimental group consistently scored

higher across most of the 46 samples. The 3.26-point mean difference represents a 24 percent improvement over the control group, highlighting the intervention's practical significance.

3.1.6 Comparative Analysis Between the Groups

This section presents a comparative analysis of pre-test and post-test scores for both control and experimental groups (N=46 each).

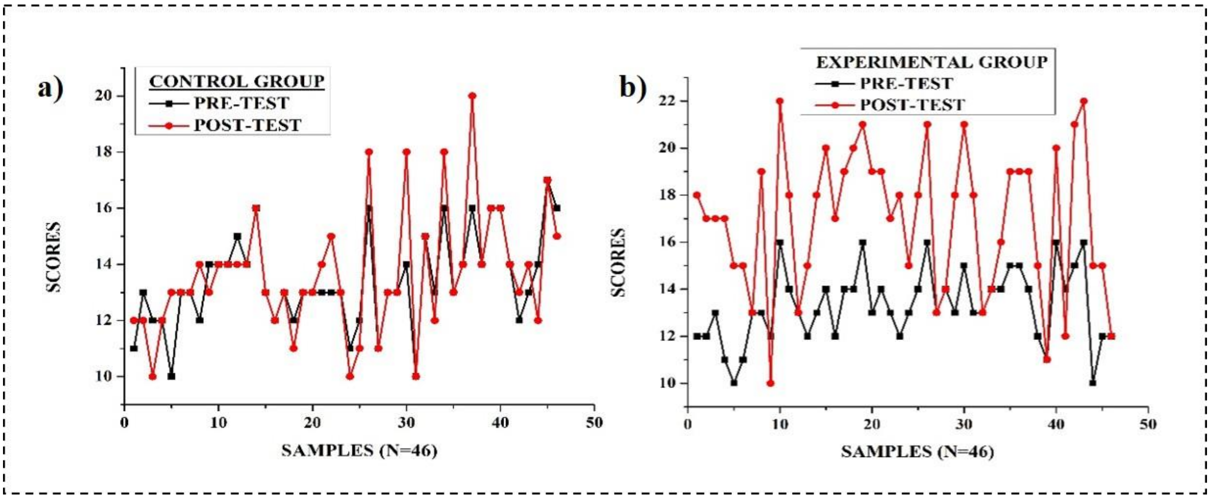

Figure 6: Pre-Test and Post- Test Results for (a) Control Group, (b) Experimental Group

As seen in Figure 6, the control group shows slight variations between pre-test and post-test scores, with scores ranging approximately from 10 to 20, indicating minimal improvement. The average difference between pre-test and post-test scores appears relatively small, suggesting that traditional assessment methods produced limited enhancement in writing skills. The experimental group, on the other hand, demonstrates a more pronounced difference between pre-test and post-test scores. While pre-test scores remain relatively consistent with the control group (ranging from approximately 10 to 16 points), the post-test scores show higher results, frequently reaching between 18 to 22 points. This indicates a substantial improvement in performance after implementing ICT-based alternative assessment methods.

These findings support the effectiveness of ICT-based alternative assessment in enhancing writing skills. The consistently higher post-test scores in the experimental group suggest that technology integration in assessment can lead to more substantial improvements in student performance. Based on the analysis, it can be concluded that the intervention positively influenced the experimental group, leading to the rejection of the null hypotheses that:

1. There is no significant difference in the improvement of writing skills between learners who receive alternative assessments and those who received traditional assessment methods.
2. The use of ICT-integrated alternative assessments does not significantly improve writing outcomes compared to traditional approaches without ICT.

3.2 Qualitative Analysis

The study employed thematic analysis [73] to identify recurring themes in qualitative data from questionnaires and interviews. Krismonica et al.'s [74] framework was adopted, which grouped writing challenges into internal and external factors, to gather learners' perspectives on PROMPTa and alternative assessment. Internal challenges included grammatical difficulties, vocabulary limitations, punctuation, spelling, and content organization, while external challenges involved understanding writing stages, motivation, time constraints, practice opportunities, and feedback availability. Coding was manually done using a hybrid approach where the framework guided initial categorization while additional themes emerged from participant's responses. The analysis highlights that these challenges are connected and can affect a learner's ability to write well.

Many learners struggled with grammatical issues (36 out of 46, or 78%) indicating reliance on dictated notes and rote memorization. Limited vocabulary (31 out of 46, or 68%) hindered self-expression indicating that learners find appropriate words to convey their thoughts, while difficulties with punctuation and spelling (35 out of 46, or 75%) were attributed to insufficient practice. Content organization (38 out of 46, or 83%) was also a major challenge as learners lacked awareness of paragraphing and logical structuring, making writing a disjointed process. External challenges were equally significant. Difficulty in understanding writing stages (43 out of 46, or 93%) indicated that learners lacked structured writing instruction, perceiving writing is a single step task rather than an iterative process. Low motivation (41 out of 46, or 89%), stemmed from writing anxiety and fear of criticism with learners describing writing as a stressful and boring. Time constraints affected 78% (36 out of 46) as writing was treated only as a means to obtain grades rather than a skill for development. Limited feedback was another recurring theme with 72% (33 out of 46) reported that teacher feedback was limited to marking errors. A strong theme was the desire for meaningful feedback with several learners frustrated by simple error corrections. Following the intervention, contrasting themes emerged. Learners valued structured nature of the feedback within PROMPTa which helped them identify strengths and weaknesses, reduce anxiety as they felt more supported and engaged when revising their work collaboratively. Structured guidance through PROMPTa's rubrics and prompts provided clear expectations, assessment tools and iterative feedback.

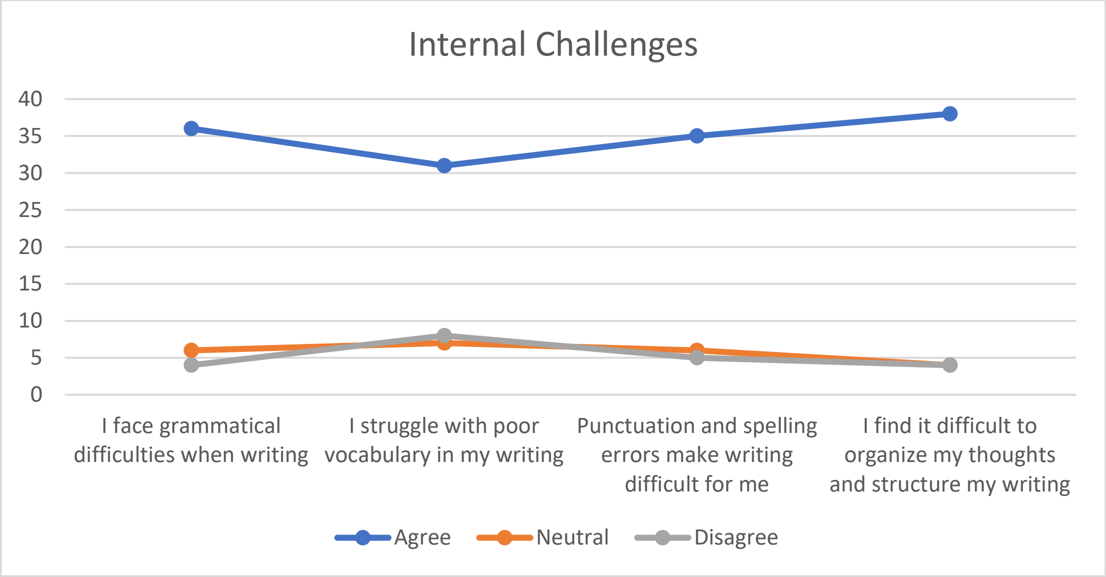

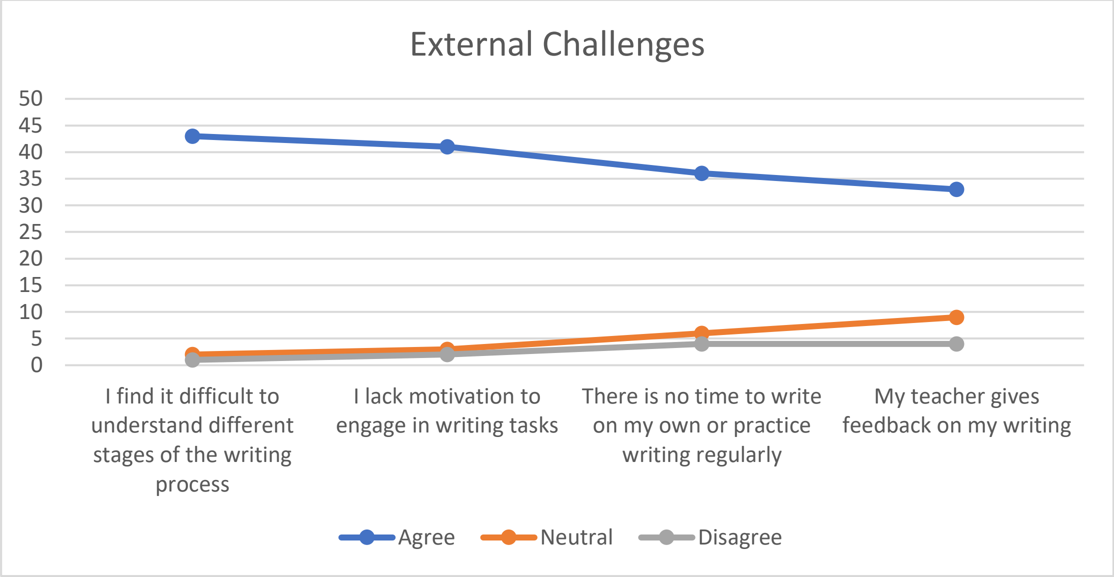

Figure 7: Writing Challenges: Internal vs. External Factors (Krismonica et al., 2021)

As seen in Figure 7, before the intervention, thematic analysis revealed that 86% of learners lacked confidence in writing and 92% were unaware of alternative assessment methods. A strong theme was their unawareness of writing expectations, making it difficult to evaluate progress. Post-intervention findings revealed greater self-awareness and control of their writing process with 78% of learners finding peer assessment and feedback very helpful, emphasizing its role in encouraging reflection and revision. One learner shared "*I really*

enjoyed reading comments on my writing from my peer. It was easy to understand and very useful as well". Another strong pre-intervention theme was the lack of exposure to ICT tools, with 79% reporting they had never used such tools in classrooms. However, post intervention responses were positive with 82% finding the prompts engaging, 88% appreciating the structured nature of PROMPTa. Anonymity emerged as a crucial factor in boosting confidence, reducing anxiety and fear of judgement, allowing them to write more freely. One learner expressed "*knowing that my work will be corrected by someone of my level and someone who does not know me made me feel less embarrassed. I wrote my heart out, whatever I wanted to express, I wrote it.*"

A strong dissatisfaction with traditional methods was a dominant pre-intervention theme with 94% learners stating that feedback was rare and limited to when someone performed really well or badly, usually to set as examples. Post intervention, learners expressed appreciation for feedback that helped them understand mistakes and improve their writing. 64% of learners found peer feedback valuable as it provided insights into common writing issues. A learner stated *"it's nice to know I am not the only one with such problems. Everyone seems to be struggling with similar problems with their writing*". Mentor and experts feedback were also highly valued. Several learners attributed their motivation and readiness to engage in the writing tasks to the inputs gained from their feedback.

The analysis reveals that internal and external challenges are deeply connected, primarily stemming from traditional assessment limitations and lack of meaningful feedback. However, integration of alternative assessment through PROMPTa addressed several challenges while encouraging greater engagement and confidence, improving overall writing development.

4. Limitations and Implications

The study acknowledges several limitations. Firstly, the research was conducted exclusively in Kohima, Nagaland and focused on undergraduate AECC students. This limited sample may introduce sample selection bias, reducing the generalizability of findings to students from other regions, courses, or educational levels and backgrounds. Also, the study is limited to a duration of eight weeks which may not be sufficient to fully capture the long-term impact of ICT-based alternative assessments. Although thematic analysis was guided by Krismonica et al.'s (2021) framework, the manual coding process may still be subject to researcher interpretation bias, as personal judgment can influence theme identification and categorization. To minimize these biases in future research, random sampling across different sample sizes, exploring different academic disciplines, and employing multiple independent coders to analyse qualitative data for greater objectivity. Expanding the study period to assess long-term impact and skill development would also strengthen the findings further.

The findings have important educational implications. It provides valuable insights into the transformative potential of ICT in assessment practices. Educators are encouraged to embrace digital platforms to make learning more interactive and learner-centered. The study also emphasizes the importance of timely, constructive feedback in developing writing and analytical skills. In conclusion, this study encourages a rethinking of traditional assessment methods and embrace technology-driven, multi-perspective feedback systems. The findings demonstrate that alternative assessments supported by ICT not only improve writing skills but also promote deeper engagement, self-reflection and collaboration among learners. As Nagaland moves towards implementing NEP 2020's vision for skill-oriented education, platforms like PROMPTa can serve as a valuable tool for promoting more inclusive, personalized, and effective learning experiences.

5. Conclusion

This study highlights the effectiveness of ICT-based alternative assessments, particularly through the PROMPTa platform, in improving writing skills in Nagaland's educational context. Findings show that learners who were involved in the scaffolded approach of peer assessment and feedback, mentor assessment and feedback, and expert assessment and feedback via PROMPTa significantly enhanced their writing proficiency. ICT-driven assessments offer a viable alternative to traditional, memorization-based evaluations, encouraging an interactive, student-centered learning environment.

The study thus, highlights the need for a shift in assessment practices, particularly in Nagaland. With the state embracing skill-based, technology-supported learning approaches, integrating ICT in assessments becomes crucial. The study identifies significant challenges such as limited infrastructure, accessibility, inadequate digital literacy among educators and students. However, the successful implementation of PROMPTa demonstrates that with structured support, ICT-driven assessments can address these gaps. The adaptability of PROMPTa, particularly it's functionality in low-connectivity environments make it practical and inclusive tool for education in regions such as Nagaland where digital access remains limited.

The multi-tiered assessment strategy addresses traditional educational limitations by moving beyond single-perspective, summative evaluations. Peers initiate critical assessment, mentors provide targeted guidance, and experts introduce professional writing standards, creating a progressive learning environment that encourages learners to continuously improve their writing skills. The findings reveal that such scaffolded assessments promote higher thinking order skills and enhance overall writing skills.

References

[1] Naveed, F., & Bhowmik, A. (2016). Definition and Techniques of Writing Skill.

[2] Hyland, K. (2003). Second language writing. Longman: Cambridge Language Education information-6233382.html

[3] Tinh, B. T. (2022). Applying peer-review checklist to improve Vietnamese EFL university students' writing skills. International Journal of Learning, Teaching and Educational Research, 21(5), 166-181. https://doi.org/10.26803/ijlter.21.5.9.

[4] Mukminatin, N. (1997). The Difference of Students' Writing Achievement across Different Courses Levels (Doctoral dissertation). Malang: State University of Malang

[5] Tierney, R. J., Soter, A., O'Flahavan, J. F., & McGinley, W. (1989). The effects of reading and writing upon thinking critically. Reading research quarterly, 134-173.

[6] Nunan, D. (2005). Practical English Language Teaching. McGraw-Hill

[7] Byrne, D. (1993). Teaching Writing Skills. Essex: Longman Group.

[8] Imane, R., & Siham, H. Investigating the Use of AI Tools to Enhance Learners' Writing Skills in the EFL Classroom (Doctoral dissertation, Kasdi Merbah Ouargla University).

[9] National Center for Educational Statistics (2012). The Nation's Report Card: Writing 2011 (NCES 2012-470). Washington, DC: National Center for Educational Statistics, Institute of Education Sciences, U.S. Department of Education

[10] ImsunaroAo, T., & Jamir, R. (2021). ELT SCENARIO AMONG THE UNDERGRADUATES IN NAGALAND: AN OVERVIEW. Literary Endeavour, 12(4).

[11] Kichu. A. (2019, July 29). English as a language but not as a subject. Nagaland Post.

[12] Linyu, K. A discussion on the problems of implementing CBCS at UG level in Nagaland. International Journal of Arts Humanities and Social Studies. 2023 Jan 1 [cited 2025 Jan 6];5(2):01–3. https://doi.org/10.33545/26648652.2023.v5.i2a.55

[13] Gardner, H. (1997). Fostering diversity through personalized education: Implications of a new understanding of human intelligence. Prospects, 27(3), 346-363.

[14] Mirror Desk. (2018, June 23). Dependency on technology reason for decline in writing skills. Eastern Mirror. https://easternmirrornagaland.com/dependence-on-technology-reason-for-decline-in-writing-skills/

[15] Brereton, J. C. (Ed.). (1995). The Origins of Composition Studies in the American College, 1875-1925. Pittsburgh: University of Pittsburgh Press.

[16] Troyka, L. Q. (2010). Simon & Schuster Handbook for Writers, 5th Ed. Toronto: Pearson Prentice Hall.

[17] Raimes, A. (1983). Methods in Teaching Writing. New York: Oxford University Press.

[18] Lasuh. (2018, June 24). 'Most undergrads still lack basic English writing skills'. Nagaland Post.

[19] Hidi, S., & Boscolo, P. (2006). Motivation and writing. Handbook of writing research, 144(157), 304-310.

[20] Richard, J. C. & Schmidt, R. (2002). Longman Dictionary of Language Teaching and Applied Linguistics (3rd Ed). London: Longman.

[21] De Smedt, F., Graham, S., & Van Keer, H. (2020). "It takes two": The added value of structured peer-assisted writing in explicit writing instruction. Contemporary Educational Psychology, 60, 101835. https://doi.org/10.1016/j.cedpsych.2019.101835

[22] Camacho, A., Alves, R. A., & Boscolo, P. (2021). Writing motivation in school: A systematic review of empirical research in the early twenty-first century. Educational psychology review, 33(1), 213-247.

[23] Lee, I. (2017). Classroom writing assessment and feedback in L2 school contexts. Singapore: Springer Singapore.

[24] Bandura, A. (1997). Self-efficacy: The exercise of control (Vol. 604). Freeman

[25] Zimmerman, B. J., & Risemberg, R. (1997). Becoming a self-regulated writer: A social cognitive perspective. Contemporary educational psychology, 22(1), 73-101. https://doi.org/10.1006/ceps.1997.0919

[26] Piaget, J. (1954). The construction of reality in the child. (M. Cook, Trans.). Basic

Books. https://doi.org/10.1037/11168-000

[27] Dewey J. Democracy and education. Columbia University Press; 2024 Dec 31. https://doi.org/10.7312/dewe21010-003

[28] Papert, S. (1980). Mindstorms: Children, Computers, and Powerful Ideas. New York, NY: Basic Books.

[29] Cuesta, P. V. N., Mayorga, J. I. M., Padilla, Y. N. P., & Mayorga, A. P. O. (2019). Alternative assessment tools for the writing skill development of EFL learners. European Scientific Journal, 15(7), 177-187.

[30] Javaherbakhsh, M. R. (2010). The Impact of Self-Assessment on Iranian EFL Learners' Writing Skill. English Language Teaching, 3(2), 213-218.

[31] Sharma, R., Jain, A., Gupta, N., Garg, S., Batta, M., & Dhir, S. K. (2016). Impact of self-assessment by students on their learning. International journal of applied and basic medical research, 6(3), 226-229. https://doi.org/10.4103/2229-516X.186961

[32] Moradan, A., & Hedayati, S. N. (2012). The impact of portfolios and conferencing on Iranian EFL learners’ writing skill. Journal of English Language Teaching and Learning, 3(8), 115-141.

[33] Punyapratheep, K. (2022). The use of portfolio in assessing English writing ability of Thai undergraduate students.

[34] Suthipiyapathra, S. (2021). Effects of Teacher-Student Conference on Paragraph Writing of EFL Students in Thailand. Universal Journal of Educational Research, 9(7), 1385-1394. https://doi.org/10.13189/ujer.2021.090705

[35] Yang, L. (2022). Focus and interaction in writing conferences for EFL writers. Sage Open, 12(1), 21582440211058200. https://doi.org/10.1177/21582440211058200

[36] Graham, S., & Perin, D. (2007). Writing next-effective strategies to improve writing of adolescents in middle and high schools.

[37] van den Bergh, H. (2015). Writing Process Theory A Functional Dynamic Approach Huub van den Bergh, Gert Rijlaarsdam, and Elke van Steendam. Handbook of writing research, 57.

[38] Ritzhaupt, A. D., & Kumar, S. (2015). Knowledge and skills needed by instructional designers in higher education. Performance Improvement Quarterly, 28(3), 51-69.https://doi.org/ 10.1002/piq.21196

[39] Al Qunayeer, H. S. (2020). Supporting postgraduates in research proposals through peer feedback in a Malaysian university. Journal of Further and Higher Education, 44(7), 956-970. https://doi.org/10.1080/0309877X.2019.1627299

[40] Bayat, M., Banihashem, S. K., & Noroozi, O. (2022). The effects of collaborative reasoning strategies on improving primary school students' argumentative decision-making skills. The Journal of educaTional research, 115(6), 349-358. https://doi.org/10.1080/00220671.2022.215

[41] Noroozi, O., & Wever, B. D. (2023). The power of peer learning: Fostering students' learning processes and outcomes. Springer Nature.

[42] Zhan, Y., Yan, Z., Wan, Z. H., Wang, X., Zeng, Y., Yang, M., & Yang, L. (2023). Effects of online peer assessment on higher-order thinking: A meta-analysis. British Journal of Educational Technology, 54(4), 817-835. https://doi.org/10.1111/bjet.13310

[43] Ketonen, L., Hähkiöniemi, M., Nieminen, P., & Viiri, J. (2020). Pathways through peer assessment: Implementing peer assessment in a lower secondary physics classroom. International Journal of Science and Mathematics Education, 18, 1465-1484.https://doi.org/10.1007/s10763-019-10030-3

[44] Kerman, N. T., Noroozi, O., Banihashem, S. K., Karami, M., & Biemans, H. J. (2024). Online peer feedback patterns of success and failure in argumentative essay writing. Interactive Learning Environments, 32(2), 614-626. https://doi.org/10.1080/10494820.2022.2093914

[45] Mangelsdorf, K. (1992). Peer reviews in the ESL composition classroom: What do the students think?. ELT journal, 46(3), 274-284. https://doi.org/10.1093/elt/46.3.274

[46] Bayraktar, A. (2013). Nature of interactions during teacher-student writing conferences, revisiting the potential effects of self-efficacy beliefs. Eurasian Journal of Educational Research, 50, 63-85.

[47] Shvidko, E. (2016). Relationship-building through embodied feedback: Teacher-student alignment in writing conferences

[48] Li, W. (2022). Exploring revision as a self-regulated process in EFL writing. Unpublished MA thesis. Canada: University of British Columbia, 10(1.0407287).

[49] Noroozi, O., & Hatami, J. (2019). The effects of online peer feedback and epistemic beliefs on students' argumentation-based learning. Innovations in Education and Teaching International. https://doi.org/10.1080/14703297.2018.1431143

[50] Pham, T. N., Lin, M., Trinh, V. Q., & Bui, L. T. P. (2020). Electronic peer feedback, EFL academic writing and reflective thinking: Evidence from a Confucian context. Sage Open, 10(1), 2158244020914554. https://doi.org/10.1177/2158244020914554

[51] Lv, X., Ren, W., & Xie, Y. (2021). The effects of online feedback on ESL/EFL writing: A meta-analysis. The Asia-Pacific Education Researcher, 30(6), 643-653. https://doi.org/10.1007/s40299-021-00594-6

[52] Zhang, R., & Zou, D. (2023). A review of research on technology-enhanced peer feedback for second language writing based on the activity theory framework. Education and Information Technologies, 28(6), 6727-6753.

[53] Zumbrunn, S., Marrs, S., & Mewborn, C. (2016). Toward a better understanding of student perceptions of writing feedback: a mixed methods study. Reading and Writing, 29, 349-370.

[54] Latifi, S., Noroozi, O., Hatami, J., & Biemans, H. J. (2021). How does online peer feedback improve argumentative essay writing and learning? Innovations in Education and Teaching International, 58(2), 195-206. https://doi.org/10.1080/14703297.2019.1687005

[55] Wu, W. C. V., Petit, E., & Chen, C. H. (2015). EFL writing revision with blind expert and peer review using a CMC open forum. Computer Assisted Language Learning, 28(1), 58-80. https://doi.org/10.1080/09588221.2014.937442

[56] Ma, Q. (2020). Examining the role of inter-group peer online feedback on wiki writing in an EAP context. Computer Assisted Language Learning, 33(3), 197-216. https://doi.org/10.1080/09588221.2018.1556703

[57] Chen, T. (2016). Technology-supported peer feedback in ESL/EFL writing classes: A research synthesis. Computer Assisted Language Learning, 29(2), 365-397. https://doi.org/10.1080/09588221.2014.960942

[58] Chen, Y. T., Li, M., Cukurova, M., & Jong, M. S. Y. (2024). Incorporation of peer-feedback into the pedagogical use of spherical video-based virtual reality in writing education. British Journal of Educational Technology, 55(2), 519-540. https://doi.org/10.1111/bjet.13376

[59] Topping, K. J. (2023). Advantages and disadvantages of online and face-to-face peer learning in higher education: A review. Education Sciences, 13(4), 326. https://doi.org/10.3390/ educsci13040326

[60] Hu, G., & Lam, S. T. E. (2010). Issues of cultural appropriateness and pedagogical efficacy: Exploring peer review in a second language writing class. Instructional science, 38, 371-394.

[61] Panadero, E., & Alonso-Tapia, J. (2013). Self-assessment: theoretical and practical connotations, when it happens, how is it acquired and what to do to develop it in our students.

[62] Winstone, N. E., Nash, R. A., Parker, M., & Rowntree, J. (2017). Supporting learners' agentic engagement with feedback: A systematic review and a taxonomy of recipience processes. Educational psychologist, 52(1), 17-37 https://doi.org/10.1080/00461520.2016.1207538

[63] Er, E., Dimitriadis, Y., & Gašević, D. (2021). A collaborative learning approach to dialogic peer feedback: a theoretical framework. Assessment & Evaluation in Higher Education, 46(4), 586-600. https://doi.org/10.1080/02602938.2020.1786497

[64] Zhu, Q., & Carless, D. (2018). Dialogue within peer feedback processes: Clarification and negotiation of meaning. Higher Education Research & Development, 37(4), 883-897. https://doi.org/10.1080/07294360.2018.1446417

[65] Goshu, K. C., & Gebremariam, H. T. (2024). Revisiting writing feedback: Using teacher-student writing conferences to enhance learners' L2 writing skills. Ampersand, 13, 100195. https://doi.org/10.1016/j.amper.2024.100195

[66] Rahmat, N. H., Thasrabiab, T., Taib, S. A., Jenal, N., Sukimin, I. S., Zamani, N. F. M., & Amir, N. (2022). Perception of difficulties and learners' reasons in academic writing: A self-imposed prophecy. International Journal of Academic Research in Business and Social Sciences, 12(10), 531-543. http://dx.doi.org/10.6007/IJARBSS/v12-i10/14870

[67] Tang, C., & Liu, Y. T. (2018). Effects of indirect coded corrective feedback with and without short affective teacher comments on L2 writing performance, learner uptake and motivation. Assessing Writing, 35, 26-40. https://doi.org/10.1016/j.asw.2017.12.002

[68] Suzuki, W., Nassaji, H., & Sato, K. (2019). The effects of feedback explicitness and type of target structure on accuracy in revision and new pieces of writing. System, 81, 135-145. https://doi.org/10.1016/j.system.2018.12.017

[69] Yusuf, Q., Jusoh, Z., & Yusuf, Y. Q. (2019). Cooperative Learning Strategies to Enhance Writing Skills among Second Language Learners. International Journal of Instruction, 12(1), 1399-1412.

[70] Nazerian, S., Abbasian, G. R., & Mohseni, A. (2021). Measurement and incorporation of ZPD scenarios in developing writing accuracy in EFL classes. Cogent Education, 8(1), 1968735. https://doi.org/10.1080/2331186X.2021.1968735

[71] Widiati, U., Rusdin, D., & Indrawati, I. (2023). The impact of AI writing tools on the content and organization of students' writing: EFL teachers' perspective. Cogent Education, 10(2). https://doi.org/ 10.1080/2331186X.2023.223

[72] Gebremariam, H. T., & Sisay, A. B. (2024). Reflective Practice in higher education institutions: Exploring teachers' reflective experiences and challenges in Ethiopian public universities. Interchange, 1-24. https://doi.org/10.1007/s10780- 024-09526-x

[73] Braun, V., Clarke, V., Hayfield, N., & Terry, G. (2019, October). Answers to frequently asked questions about thematic analysis.

[74] Krismonica, D., Harmanto, B., Mustikawati, D. A., & Maghfiroh, A. (2021). A Study of Students' Errors on Paragraph Writing. English Focus: Journal of English Language Education, 4(2), 112-124. https://doi.org/10.24905/efj.v4i2.10

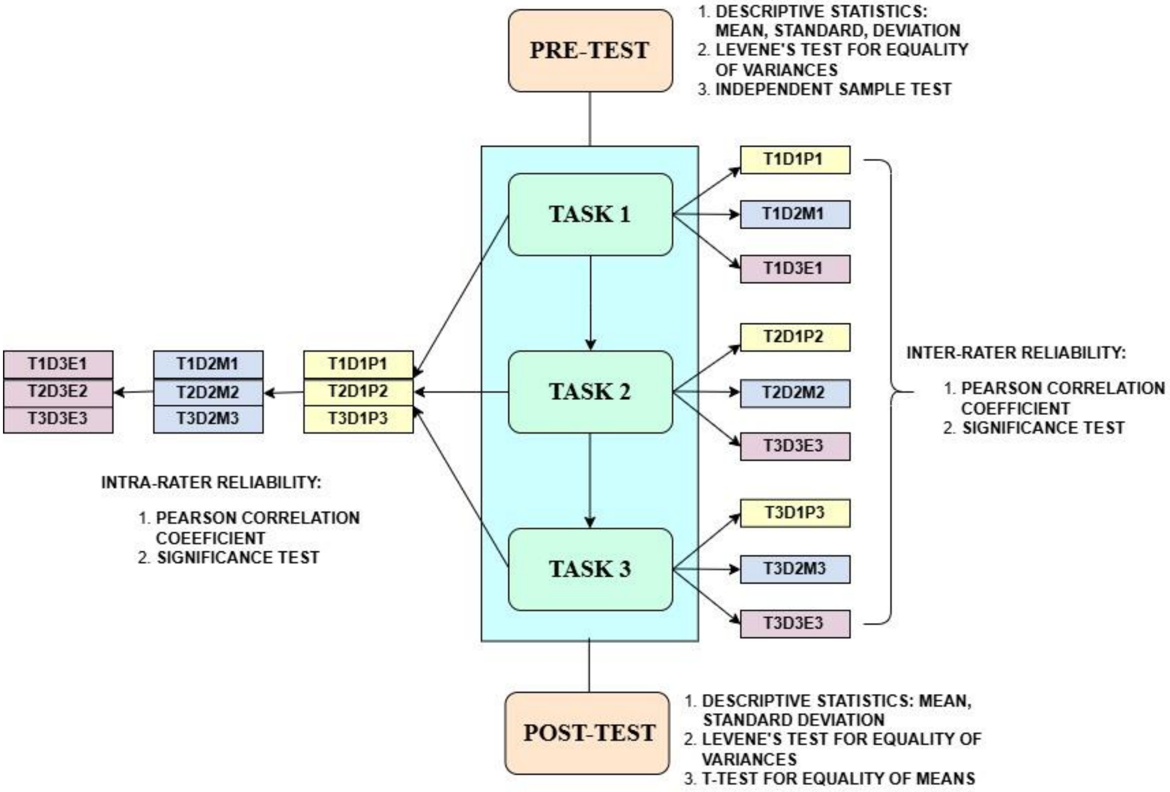

Figure 1: Quantitative Analysis
Note: T= Tasks, D=Drafts, P=Peer-assessment, M=Mentor Assessment and Feedback, E=Expert Assessment and Feedback

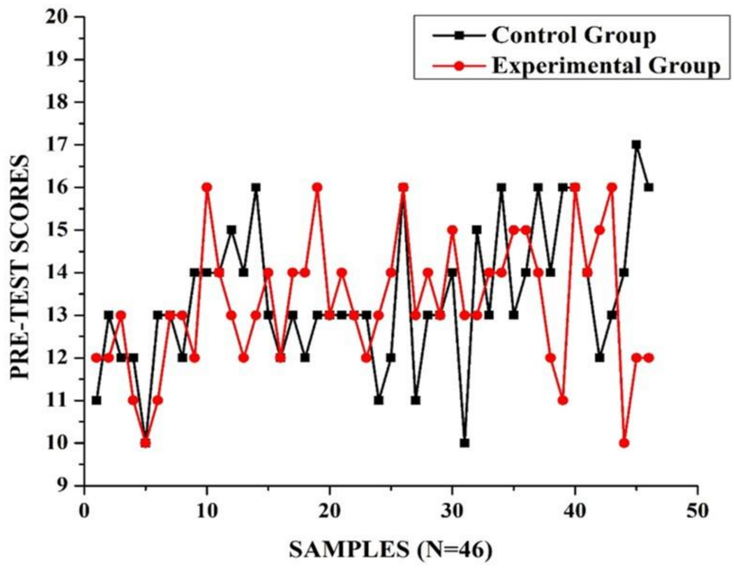

Figure 2: Graph for Control Group and Experimental Group (Pre-Test)

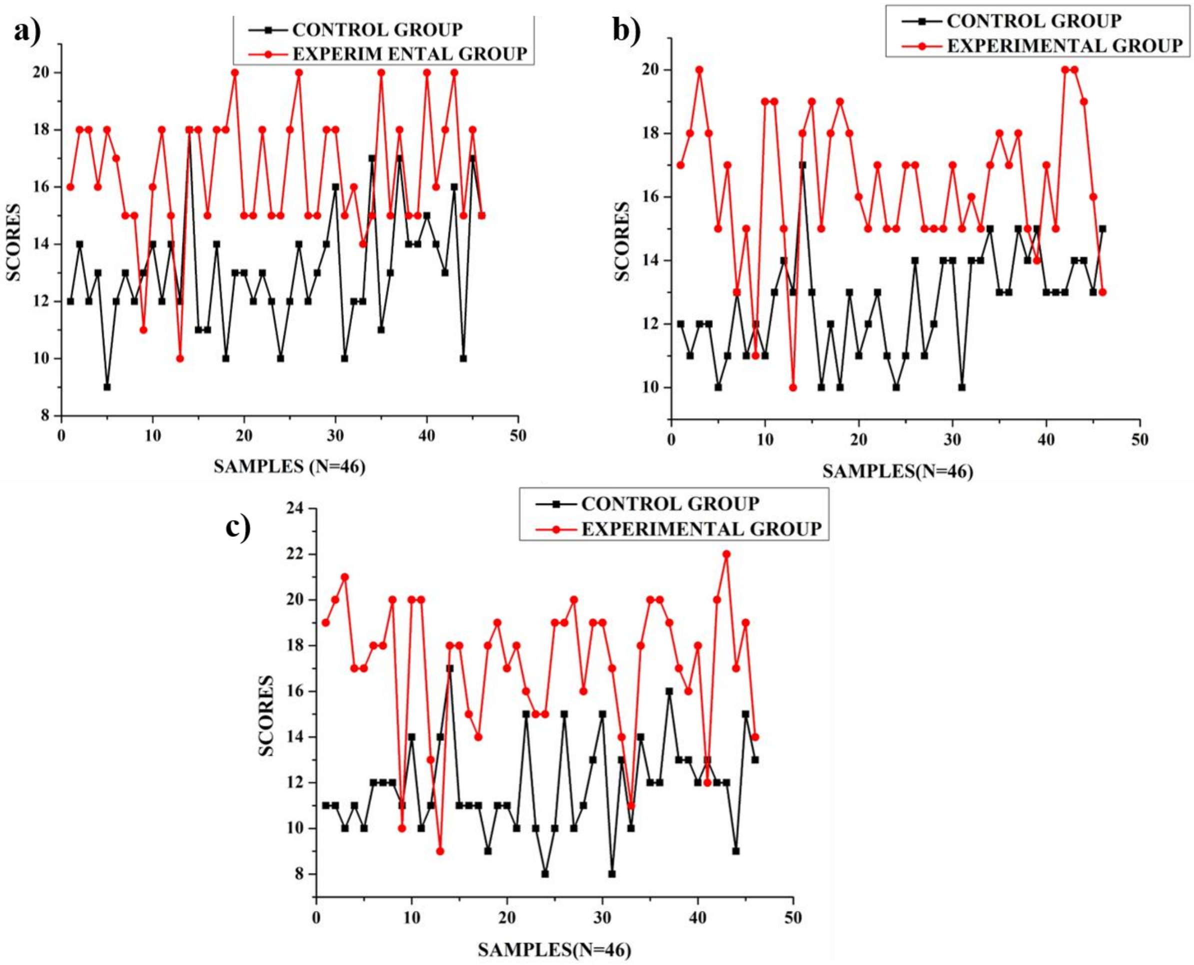

Figure 3: Intra-rater reliability estimates for control group and experimental group: (a) T1D2=Control Group, T1D2M1=Experimental Group, (b) T2D2=Control Group, T2D2M2= Experimental Group and (c) T3D2=Control Group, T3D2M3=Experimental Group

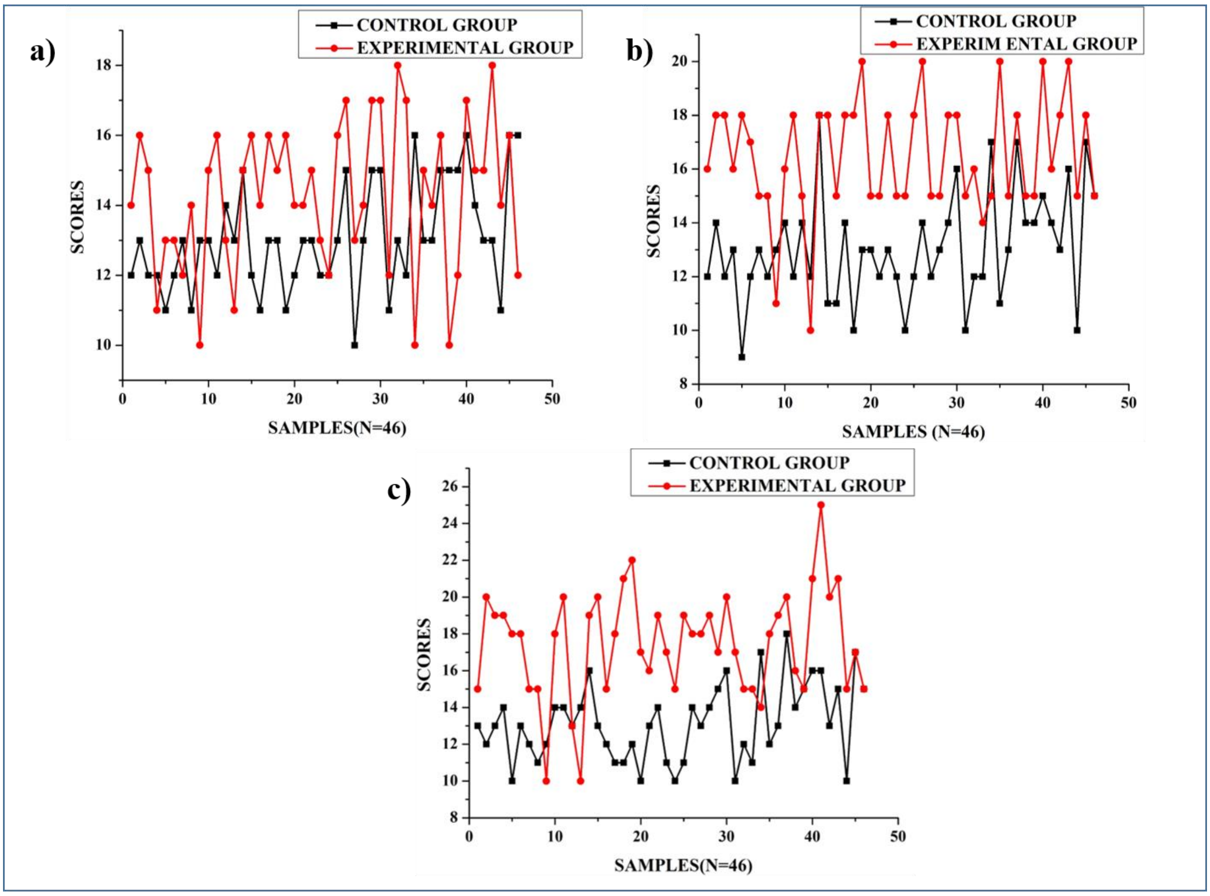

Figure 4: Inter-reliability for both control and experimental group, (a) T1D1=Control Group, T1D1P1=Experimental Group, (b) T1D2=Control Group, T1D2M1= Experimental Group, and (c) T1D3=Control Group, T1D3E1=Experimental Group

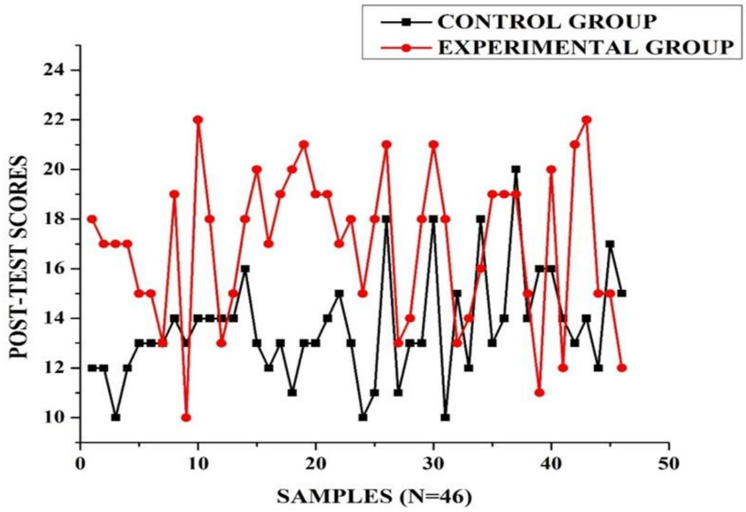

Figure 5: Graph for Control Group and Experimental Group (Post-Test)

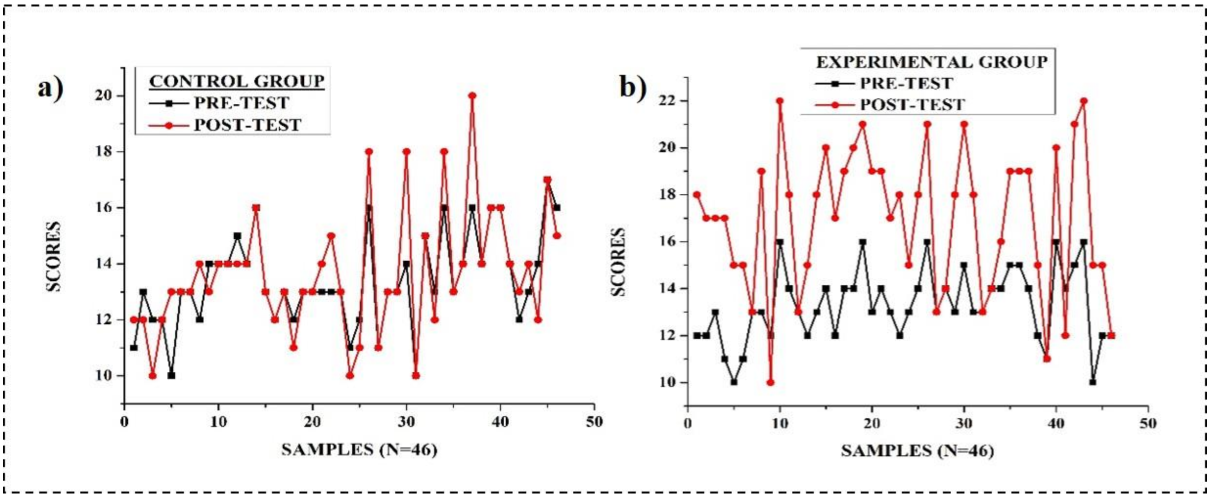

Figure 6: Pre-Test and Post- Test Results for (a) Control Group, (b) Experimental Group

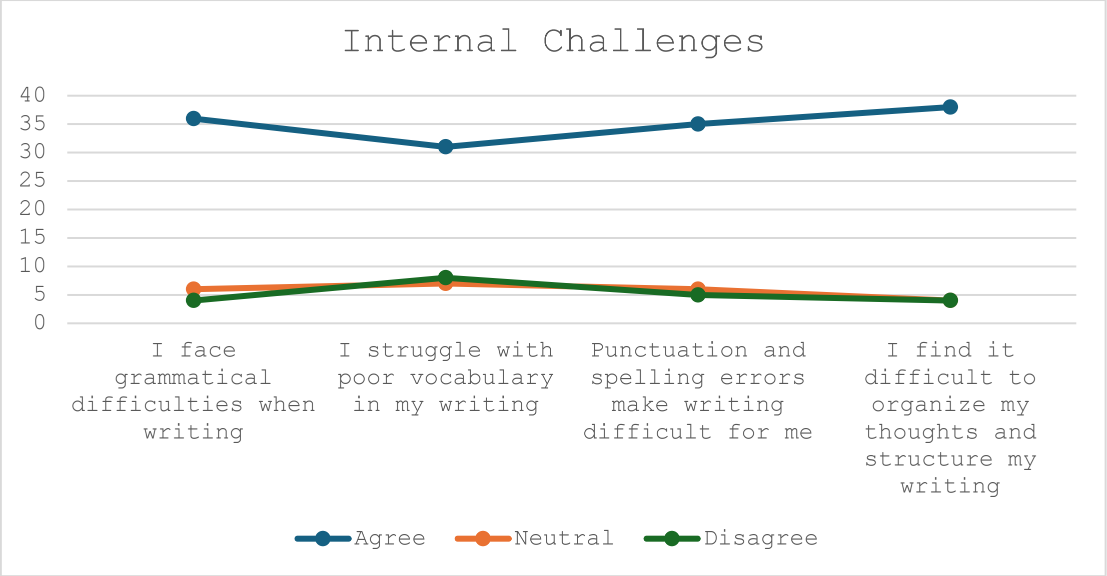

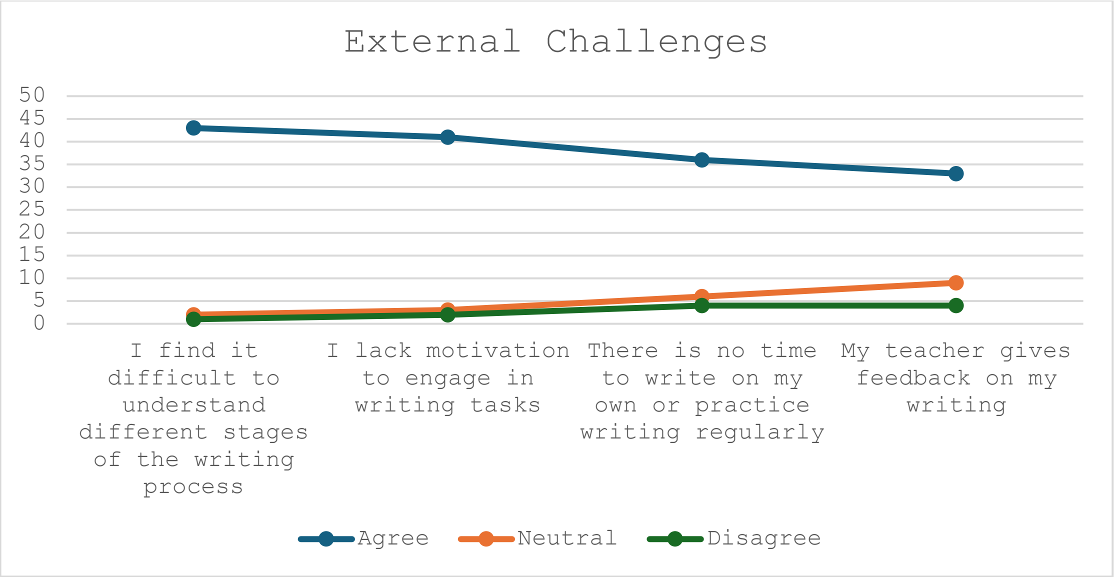

Figure 7: Writing Challenges: Internal vs. External Factors (Krismonica et al., 2021)

Data

Click here to access/download
Supporting Information
DATA .xlsx
