# Supplementary material for: PROMPTa—A multifaceted assessment approach to develop writing skills
Source: PLoS One. 2025 Dec 2;20(12):e0337342. doi: 10.1371/journal.pone.0337342 (PMC12671783; doi:10.1371/journal.pone.0337342)
Supplement: Attachment — Submitted filename: Revision Text.docx [file pone.0337342.s002.docx]

**Abstract Revision Notes**

1. Introduction: already present, but too general.
2. Methods: present, but need to be summarized and written more formally.
3. Results: quite informative, but can be condensed.
4. Conclusion: the conclusion is still weak and too descriptive.
5. Structure the abstract explicitly in the framework: Background – Purpose – Methods – Results – Conclusion.
6. PROMPTa needs to be explained concisely from the beginning as a tool-based ICT-integrated multifaceted assessment framework.
7. Avoid too many numbers in the abstract; just the main data and significant differences.
8. Need to add one sentence about the theoretical or practical contribution of the findings, for example:
9. "These findings suggest that multifaceted peer-assisted ICT-based assessment tools like PROMPTa may significantly improve writing instruction strategies in higher education."
10. Avoid excessive statistical detail
11. Add main contributions/implications

**Keyword Revision Notes**

1. Too many or too specific keywords tend to interfere with the indexing process and relevance of scientific search engines.
2. Combine closely related terms or reduce redundancy.
3. Avoid overly long phrases such as “Peer Assessment and Feedback”. Use a concise form that is commonly used in international literature.
4. Some keywords overlap too much (e.g.: Peer Feedback, Mentor Feedback, and Expert Feedback can be combined as Multisource Feedback).
5. The main focus should be reflected, for example: Writing Skills Development, Alternative Assessment, ICT Integration.
6. Recommended Keywords: 1) PROMPTa, Alternative Assessment, Peer and Expert Feedback, Writing Skills Development, ICT Integration, Higher Education. 2). Keywords: PROMPTa, Alternative Writing Assessment, Multisource Feedback, ICT in Education, Academic Writing.

**Preface Revision Notes**

1. Too long and mixed between theoretical context, local, and study objectives.
2. Not explicitly divided into important elements: background – gap – urgency – objectives.
3. Suggestions Divide the introduction into 4 main paragraphs: 1). Global context: the importance of writing skills in the digital era and global challenges. 2). Local context: conditions in Nagaland and its challenges. 3). Theoretical gap: limitations of traditional assessment, the need for alternative approaches (PROMPTa). 4). Purpose: formulation of the problem and contribution of the study.
4. Sentences such as "The current education system fails to empower learners." sound too normative or subjective. International journals prefer neutral and data-based diction.
5. Some sections are repetitive, especially stating that the system in Nagaland is still traditional.
6. The introduction has not explicitly stated the literature gap, which is a must in a reputable journal.
7. There needs to be one explicit sentence at the end that formulates the purpose of the study and its contribution.

**Revision Notes: Theoretical Framework & Literature Review**

1. The description of the theory seems descriptive and fragmentary, not critically linked to each other or to the focus of the study.
2. Combine the theories into an integrated conceptual framework and state how this theory guided the study design (e.g., how SCT, peer feedback, technology, writing outcomes).
3. SCT, Constructivism, and Constructionism are described separately without a clear bridge.
4. Create an integrative paragraph that brings the theories together into a conceptual framework that supports the research design.

**Research Methodology Revision Notes**

1. It is called "mixed-methods," but it does not explain the type of approach (e.g., sequential explanatory, convergent, or exploratory sequential) and how the quantitative and qualitative data were integrated.
2. Add the type of mixed-methods design and how the qualitative and quantitative results were integrated.
3. It does not explain whether this is a true experiment or a quasi-experiment (because it is likely not random assignment).
4. Clarify that this is a quasi-experimental design if the assignment of participants is not random.
5. It does not explain the number of participants, sampling technique, institutions, and important characteristics (gender, age, program of study).
6. Add a detailed description of the participants, number, sampling method, and institution of origin.
7. The explanation is quite complete but is written in an informal narrative format and is somewhat repetitive.
8. Use subheadings such as Week-by-Week Intervention Plan or Instructional Phases, and focus on: objectives, activities, media, and evaluation.
9. No mention of the type of test, assessment criteria (rubric), validity, and reliability.
10. Add a description of the pre/post-test instrument and perception measurement tools (questionnaires and interviews).
11. No statement of ethical approval or informed consent.
12. Add a statement that ethical approval has been obtained.

**Revision Notes for the "Limitations and Implications of the Study" Section**

1. Limitations are written narratively and mixed without systematic grouping (eg: methodology, context, duration, analysis).
2. Classify limitations into several categories such as: sampling, intervention duration, and data analysis, then explain them in a concise and focused manner.
3. Implications are too focused on practice (implementation), whereas Scopus journals usually expect contributions to existing theories or models.
4. Add how these findings contribute to previous theoretical frameworks, for example alternatives.

**Revision Notes for Conclusions**

1. Phrases such as “confirms effectiveness,” “demonstrates significant improvement,” or “demonstrates that scaffolded assessment approaches can…” sound too strong for a study with limited context.
2. Use academic language that is tentative and based on the context of the findings, such as: “suggests,” “indicates potential,” “within this context.”
3. The conclusion focuses only on the context and practical outcomes without explaining the contribution to theory or previous literature.
4. Add one reflective paragraph that links the study’s findings to strengthening or expanding a theoretical framework, such as formative assessment theory, peer feedback, or edtech for equity.
5. There is no explicit statement about directions or recommendations for further research.
6. Add one sentence about how the study could be continued: longer duration, different contexts, or a longitudinal approach.
7. Some phrases sound descriptive or narrative rather than analytical and academic.
8. Use a formal, concise style that is based on a synthesis of findings rather than a re-description of the research process.
