# Supplementary material for: PROMPTa—A multifaceted assessment approach to develop writing skills
Source: PLoS One. 2025 Dec 2;20(12):e0337342. doi: 10.1371/journal.pone.0337342 (PMC12671783; doi:10.1371/journal.pone.0337342)
Supplement: Attachment — Submitted filename: Response to Reviewers.docx [file pone.0337342.s004.docx]

**Response to Reviewers**

Dear Editors and Reviewers,

Please find below our thorough and thoughtful responses to the invaluable feedback the Editors and Reviewers provided. Each comment has been carefully considered, and we have taken diligent measures to address every item raised. Our commitment to improving the quality of this manuscript is evident in the revisions we have made.

On behalf of all the authors, I express my sincere gratitude to the Reviewers and the esteemed Editors for their constructive input and valuable suggestions. Their guidance has undoubtedly played a significant role in refining this piece of work. We are genuinely appreciative of this opportunity to enhance our research, and we eagerly look forward to sharing the enhanced and polished version with the esteemed readership of the journal in the near future.

**E: Editor	R: Reviewer	C: Comment(s)**

| **Comments** | **Authors’ response** | **Changes made** | **Reference in Manuscript**  **(Page Numbers)** |
| --- | --- | --- | --- |
| E.C.1. Based on the decisions given by reviewers, this paper requires MINOR REVISION. Please look through the comments and amend accordingly. Thank you. | E.C.1. Thanks for recommending MINOR REVISION. We will implement the necessary improvements based on the Reviewers' comments. | The improvements have been incorporated throughout the paper. | The paper has been revised as required to address the comments. |
| **1. Abstract Revision Notes**  R1C1.1. Introduction: already present, but too general.  R1C1.2. Methods: present but need to be summarized and written more formally.  R1C1.3. Results: quite informative but can be condensed.  R1C1.4. Conclusion: The conclusion is still weak and too descriptive.  R1C1.5. Structure the abstract explicitly in the framework: Background – Purpose – Methods – Results – Conclusion.  R1C1.6. PROMPTa needs to be explained concisely from the beginning as a tool-based ICT-integrated multifaceted assessment framework.  R1C1.7. Avoid too many numbers in the abstract, just the main data and significant differences.  R1C1.8. Need to add one sentence about the theoretical or practical contribution of the findings, for example:  R1C1.9. “These findings suggest that multifaceted peer-assisted ICT-based assessment tools like PROMPTa may significantly improve writing instruction strategies in higher education.”  R1C1.10. Avoid excessive statistical detail  R1C1.11. Add main contributions/ implications | R1C1.1. Thank you for your insightful observation and for sharing your thoughts to improve its quality.  R1C1.2. Thank you for your observation. Changes have been made accordingly.  R1C1.3. Thank you for highlighting this point. It helps us see where improvement is needed.  R1C1.4. Your constructive critique adds significant value. Changes have been made to reflect your suggestions.  R1C1.5. Thank you for your guidance. Changes have been integrated to improve clarity and coherence.  R1C1.6. We appreciate this important clarification.  R1C1.7. Thank you for pointing this out.  R1C1.8. Thank you for your valuable suggestion.  R1C1.9. Thank you. The sentence has been incorporated into the abstract.  R1C1.10. It is a much-appreciated comment.  R1C1.11. Thank you for highlighting this. A final sentence has been added to complete the abstract meaningfully. | R1C1.1. The introduction in the abstract has been revised in response to the reviewer’s comment.  R1C1.2. The methods section has been summarised and written in a more formal style.  R1C1.3. The results have been condensed.  R1C1.4. Conclusion has been strengthened.  R1C1.5. The abstract has been restructured accordingly.  R1C1.6. Changes have been made to ensure a better understanding of PROMPTa’s function.  R1C1.7. Only the most relevant figures, which reflect significant differences and outcomes, have been highlighted.  R1C1.8. A sentence has been added to highlight the study’s contribution to assessment practice and its relevance for writing instruction.  R1C1.9. The sentence has been incorporated into the abstract.  R1C1.10. The abstract has been revised to focus only on key outcomes.  R1C1.11. Main contributions and implications have been included. | R1C1.1. The revision can be seen in the following page, Page No. 2  R1C1.2. The revision can be seen in the following page, Page No. 2  R1C1.3. The revision can be seen in the following page, Page No.2  R1C1.4. The revision can be seen on the following page, Page No.2  R1C1.5. The revision can be seen in the following page, Page No. 2  R1C1.6. The revision can be seen in the following page, Page No. 2  R1C1.7. The revision can be seen in the following page, Page No. 2  R1C1.8. The revision can be seen in the following page, Page No. 2  R1C1.9. The revision can be seen in the following page, Page No. 2  R1C1.10. The revision can be seen in the following page, Page No. 2  R1C1.11. The revision can be seen in the following page, Page No. 2 |
| **2. Keyword Revision Notes**  R1C2.1. Too many or too specific keywords tend to interfere with the indexing process and relevance of scientific search engines.  R1C2.2. Combine closely related terms or reduce redundancy.    R1C2.3. Avoid overly long phrases such as “Peer Assessment and Feedback”. Use a concise form that is commonly used in international literature.  R1C2.4. Some keywords overlap too much (e.g.: Peer Feedback, Mentor Feedback, and Expert Feedback can be combined as Multisource Feedback).  R1C2.5. The main focus should be reflected, for example: Writing Skills Development, Alternative Assessment, ICT Integration.  R1C2.6. Recommended Keywords: 1)  PROMPTa, Alternative Assessment, Peer and Expert Feedback, Writing Skills Development, ICT Integration, Higher Education. 2). Keywords: PROMPTa, Alternative Writing Assessment, Multisource Feedback, ICT in Education, Academic Writing. | R1C2.1.Thank you for your relevant observation.  R1C2.2. We appreciate the suggestion regarding redundancy. The changes have been addressed.  R1C2.3. Thank you for this helpful recommendation.  R1C2.4. Thank you for the helpful recommendation.  R1C2.5. Thank you for this valuable insight.  R1C2.6. Thank you for the recommended keywords. We have revised the keywords accordingly. | R1C2.1. We have reduced the number of keywords to enhance the search engine performance.  R1C2.2. Redundancy has been addressed.  R1C2.3. Overly long phrases have been replaced with more concise words.  R1C2.4. Overlapping terms have now been consolidated under the broader term “Multisource Feedback” to improve focus and precision.  R1C2.5. The revised keywords now more accurately reflect the study's core focus.  R1C2.6. Recommended keywords have been added. | R1C2.1. The revision can be seen in the following page, Page No. 2  R1C2.2. The revision can be seen in the following page, Page No. 2  R1C2.3. The revision can be seen in the following page, Page No. 2  R1C2.4. The revision can be seen in the following page, Page No. 2  R1C2.5. The revision can be seen in the following page, Page No. 2  R1C2.6. The revision can be seen in the following page, Page No. 2 |
| **3. Keyword Revision Notes**  R1C3.1. Too long and mixed between theoretical context, local, and study objectives.    R1C3.2. Not explicitly divided into important elements: background – gap – urgency – objectives.  R1C3.3. Suggestions Divide the introduction into 4 main paragraphs: 1). Global context: the importance of writing skills in the digital era and global challenges. 2). Local context: conditions in Nagaland and its challenges. 3). Theoretical gap: limitations of traditional assessment, the need for alternative approaches (PROMPTa). 4). Purpose: formulation of the problem and contribution of the study.  R1C3.4. Sentences such as “The current education system fails to empower learners.” sound too normative or subjective. International journals prefer neutral and data-based diction.  R1C3.5. Some sections are repetitive, especially stating that the system in Nagaland is still traditional.    R1C3.6. The introduction has not explicitly stated the literature gap, which is a must in a reputable journal.  R1C3.7. There needs to be one explicit sentence at the end that formulates the purpose of the study and its contribution. | R1C3.1. Thank you for the feedback. The introduction has been revised for better clarity.  R1C3.2. We appreciate this guidance as it improves the clarity of the study.  R1C3.3. Thank you for the structured suggestion. It helped identify some oversights and opportunities for improvement.  R1C3.4. We acknowledge this concern and are grateful for the observation.  R1C3.5. Thank you for pointing out the repetition.  R1C3.6. Thank you for this important note.  R1C3.7. We appreciate this suggestion. | R1C3.1. The introduction has been shortened and organized for better clarity.  R1C3.2. The introduction is now clearly divided into the suggested elements.  R1C3.3. We have reorganized the introduction into four distinct paragraphs as recommended.  R1C3.4. Normative language has been revised to reflect a more neutral, evidence-based tone.  R1C3.5. Redundant statements have been removed for conciseness and clarity.  R1C3.6. The revised introduction now includes a clear articulation of the literature gap.  R1C3.7. A concluding sentence has been added to clearly state the study’s aim and its contribution. | R1C3.1. The revision can be seen in the following page, Page No. 3- 5  R1C3.2. The revision can be seen in the following page, Page No. 3- 5  R1C3.3. The revision can be seen in the following page, Page No. 3- 5  R1C3.4. The revision can be seen in the following page, Page No. 4  R1C3.5. The revision can be seen in the following page, Page No. 3- 5  R1C3.6. The revision can be seen in the following page, Page No. 3- 5  R1C3.7. The revision can be seen in the following page, Page No. 5 |
| **4. Revision Notes: Theoretical Framework & Literature Review**  R1C4.1. The description of the theory seems descriptive and fragmentary, not critically linked to each other or to the focus of the study.  R1C4.2. Combine the theories into an integrated conceptual framework and state how this theory guided the study design (e.g., how SCT, peer feedback, technology, writing outcomes).  R1C4.3. SCT, Constructivism, and Constructionism are described separately without a clear bridge.  R1C4.4. Create an integrative paragraph that brings the theories together into a conceptual framework that supports the research design.  **5. Research Methodology Revision Notes**  R1C5.1. It is called “mixed methods,” but it does not explain the type of approach (e.g., sequential explanatory, convergent, or exploratory sequential) and how the quantitative and qualitative data were integrated.  R1C5.2. Add the type of mixed-methods design and how the qualitative and quantitative results were integrated.  R1C5.3. It does not explain whether this is a true experiment or a quasi-experiment (because it is likely not random assignment).  R1C5.4. Clarify that this is a quasi-experimental design if the assignment of participants is not random.  R1C5.5. It does not explain the number of participants, sampling technique, institutions, and important characteristics (gender, age, program of study).  R1C5.6. Add a detailed description of the participants, number, sampling method, and institution of origin.  R1C5.7. The explanation is quite complete but is written in an informal narrative format and is somewhat repetitive.  R1C5.8. Use subheadings such as Week-by-Week Intervention Plan or Instructional Phases, and focus on: objectives, activities, media, and evaluation.  R1C5.9. No mention of the type of test, assessment criteria (rubric), validity, and reliability.  R1C5.10. Add a description of the pre/post-test instrument and perception measurement tools (questionnaires and interviews).  R1C5.11. No statement of ethical approval or informed consent.  R1C5.12. Add a statement that ethical approval has been obtained.  **6. Revision Notes for the Limitations and Implications of the Study Section** R1C6.1. Limitations are written narratively and mixed without systematic grouping (eg: methodology, context, duration, analysis).  R1C6.2. Classify limitations into several categories such as sampling, intervention duration, and data analysis, then explain them in a concise and focused manner.  R1C6.3. Implications are too focused on practice (implementation), whereas Scopus journals usually expect contributions to existing theories or models.  R1C6.4. Add how these findings contribute to previous theoretical frameworks, for example alternatives.  **7. Revision Notes for Conclusions** R1C7.1. Phrases such as “confirms effectiveness,” “demonstrates significant improvement,” or “demonstrates that scaffolded assessment approaches can…” sound too strong for a study with limited context.  R1C7.2. Use academic language that is tentative and based on the context of the findings, such as: “suggests,” “indicates potential,” “within this context.”  R1C7.3. The conclusion focuses only on the context and practical outcomes without explaining the contribution to theory or previous literature.  R1C7.4. Add one reflective paragraph that links the study’s findings to strengthening or expanding a theoretical framework, such as formative assessment theory, peer feedback, or edtech for equity.  R1C7.5. There is no explicit statement about directions or recommendations for further research.  R1C7.6. Add one sentence about how the study could be continued: longer duration, different contexts, or a longitudinal approach.  R1C7.7. Some phrases sound descriptive or narrative rather than analytical and academic.  R1C7.8. Use a formal, concise style that is based on a synthesis of findings rather than a re-description of the research process. | R1C4.1. Thank you for the keen observation. It helps strengthen the study conceptually.  R1C4.2. We appreciate this suggestion, offering critical depth and coherence to the study.  R1C4.3. Thank you for pointing this out.  R1C4.4. Thank you for your helpful suggestion for the enhancement of the quality of the paper.  R1C5.1. Thank you for highlighting this. It is highly appreciated.  R1C5.2. We appreciate the suggestion.  R1C5.3. Thank you for pointing out this need for clarification.  R1C5.4. Thank you. It is acknowledged.  R1C5.5. Thank you for this feedback.  R1C5.6. Thank you for pointing out a highly relevant oversight.  R1C5.7. Thank you for pointing this out.  R1C5.8. We appreciate this helpful suggestion.  R1C5.9. Thank you for this important point.  R1C5.10. Thank you. It is acknowledged.  R1C5.11. Thank you for this important oversight.  R1C5.12. Appreciate the reminder.  R1C6.1. Thank you for the feedback.  R1C6.2. We appreciate the suggestion.    R1C6.3. Thank you for the valuable insight.  R1C6.4. Thank you for the suggestion.  R1C7.1. Thank you for pointing this out.  R1C7.2. Thank you for the suggestion.  R1C7.3. Your keen observation is highly appreciated. It contributes meaningfully to both the conceptual and practical depth of this work.  R1C7.4. Thank you for your observations, which opens important avenues for refinement.  R1C7.5. Thank you for pointing that out.  R1C7.6. Thank you for your valuable suggestion.  R1C7.7. Thank you for your observation.  R1C7.8. Thank you for your careful attention to detail. | R1C4.1. The theoretical discussion has been revised to be more cohesive and directly linked to the study focus.  R1C4.2. The revised section presents an integrated framework showing how each theory informed the study design.  R1C4.3. These theories are now conceptually connected and positioned in relation to peer feedback and ICT-supported writing.  R1C4.4. A new paragraph has been added to synthesize the theoretical foundations into one coherent framework.  R1C5.1 The revised methods section now specifies the design as a sequential explanatory mixed-methods approach.  R1C5.2. We have clarified how the two data types were integrated during the interpretation process.  R1C5.3. It has now been stated that the study followed a quasi-experimental design due to the lack of random assignment.  R1C5.4. The methods section now clearly labels the design as quasi-experimental with justification.  R1C5.5. We have now included detailed participant information, including numbers, demographics, and sampling technique.  R1C5.6. The revised version includes the suggested descriptions.  R1C5.7. The narrative has been edited for conciseness and formal academic tone.  R1C5.8. The intervention section now utilises subheadings, such as “Instructional Phases,” and follows a structured format.  R1C5.9. We have added the missing details as pointed.  R1C5.10. The revised section now includes the suggested descriptions.  R1C5.11. We have now included a statement clarifying that ethical procedures and informed consent were ensured.  R1C5.12. The manuscript now clearly states that ethical approval was obtained.  R1C6.1. The limitations have been reorganized to improve structure.  R1C6.2. The limitations are now categorized under sampling, intervention design, and analysis to ensure clarity and focus.  R1C6.3. The implications section now includes both theoretical and practical contributions.  R1C6.4. The revised version connects findings to the existing frameworks.  R1C7.1. Strong phrases have been replaced.  R1C7.2. The changes have been made to use academic and tentative language.  R1C7.3. The conclusion summarises the study's contribution to the theory, as well as its relation to previous literature.  R1C7.4. A reflective paragraph has been included, linking the study’s findings to the strengthening of the theoretical framework.  R1C7.5. A statement suggesting directions for future studies has been added at the end of the conclusion.  R1C7.6. A sentence recommending longitudinal studies and application in diverse educational contexts has been included.  R1C7.7. The conclusion has been rewritten in a more analytical and academic tone.  R1C7.8. The conclusion is now more concise and based on the synthesis of key insights. | R1C4.1. The revision can be seen in the following page, Page No. 7  R1C4.2. The revision can be seen in the following page, Page No. 7-9  R1C4.3. The revision can be seen in the following page, Page No. 7-9  R1C4.4. The revision can be seen in the following page, Page No. 9  R1C5.1 The revision can be seen in the following page, Page No. 12  R1C5.2. The revision can be seen in the following page, Page No. 12  R1C5.3. The revision can be seen in the following page, Page No. 12  R1C5.4. The revision can be seen in the following page, Page No. 12  R1C5.5. The revision can be seen in the following page, Page No. 13  R1C5.6. The revision can be seen in the following page, Page No. 13  R1C5.7. The revision can be seen in the following page, Page No. 13  R1C5.8. The revision can be seen in the following page, Page No. 13-14  R1C5.9. The revision can be seen in the following page, Page No. 14-15  R1C5.10. The revision can be seen in the following page, Page No. 14-15  R1C5.11. The revision can be seen in the following page, Page No. 13  R1C5.12. The revision can be seen in the following page, Page No. 13  R1C6.1. The revision can be seen in the following page, Page No. 29  R1C6.2. The revision can be seen in the following page, Page No. 29  R1C6.3. The revision can be seen in the following page, Page No. 29-30  R1C6.4. The revision can be seen in the following page, Page No. 30  R1C7.1. The revision can be seen on the following page,  Page No. 30  R1C7.2. The revision can be seen in the following page, Page No. 31  R1C7.3. The revision can be seen in the following page, Page No. 31  R1C7.4. The revision can be seen in the following page, Page No. 31  R1C7.5. The revision can be seen in the following page, Page No. 31  R1C7.6. The revision can be seen in the following page, Page No. 31  R1C7.7. The revision can be seen in the following page, Page No. 31  R1C7.8. The revision can be seen in the following page, Page No. 31 |
| R2C1. The PROMPTa tool presents an innovative approach by combining peer, mentor, and expert feedback. | R2C1. Thank you for the positive recognition of the design of PROMPTa. It is highly encouraging. | R2C1. (NIL) | R2C1. (NIL) |
| R2C2. The research design is rigorous, and statistical methods are appropriately used.  R2C3. The thematic analysis provides useful insight into learner challenges and the effectiveness of the intervention.  R2C4. The manuscript is well contextualized within India’s National Education Policy (NEP 2020) and existing challenges in language instruction.  R2C5. Suggestions for Improvement:  R2C6. Reduce Redundancy: Consider condensing sections of the introduction and literature review that repeat similar arguments about writing challenges and assessment limitations.  R2C7. Technical Description of PROMPTa: More details on the technical framework, user interface, and accessibility features of the PROMPTa platform would be helpful.  R2C8. Examples of Feedback: Providing examples or screenshots of feedback (especially anonymized peer or expert comments) would strengthen the reader’s understanding.  R2C9. Long-Term Impact: While the 8-week intervention is sufficient for initial analysis, a brief note on follow-up plans (or suggestions) for long-term tracking would improve the impact discussion.  R2C10. Language Polish: Minor grammatical edits are needed to streamline some long paragraphs. | R2C2. We sincerely appreciate your positive feedback.  R2C3. Thank you for this encouraging note.  R2C4. We thank you for acknowledging the contextual grounding of the study.  R2C5. Your keen observation is deeply appreciated. It points the work in the right direction for further refinement  R2C6. Thank you for your detailed observations.  R2C7. Your comments reflect both care and expertise.  R2C8. Thank you for pointing this out.  R2C9. Thank you for this guidance.  R2C10. Thank you for highlighting this. | R2C2. (NIL)  R2C3. (NIL)  R2C4. (NIL)  R2C5. (NIL)  R2C6. Repetitive sections have been condensed to improve focus and readability.  R2C7. As per the suggestion, a clearer explanation of PROMPTa’s structure, interface, and accessibility features has been added.  R2C8. As per the suggestions, screenshots of the feedback have been attached.  R2C9. A brief note on plans for future longitudinal tracking has been added in the discussion section.  R2C10. Grammatical issues have been corrected, and long paragraphs have been refined for improved flow. | R2C2. (NIL)  R2C3. (NIL)  R2C4. (NIL)  R2C5. (NIL)  R2C6. The revision can be seen in the following page, Page No.3- 5  R2C7. The revision can be seen in the following page, Page No. 14-16  R2C8. The revision can be seen in the following pages,  Page No. 24, 25, 26.  R2C9. The revision can be seen in the following page, Page No. 29-30  R2C10. The revision is evident throughout the entire paper. |
