# Supplementary material for: PROMPTa—A multifaceted assessment approach to develop writing skills
Source: PLoS One. 2025 Dec 2;20(12):e0337342. doi: 10.1371/journal.pone.0337342 (PMC12671783; doi:10.1371/journal.pone.0337342)
Supplement: Attachment — Submitted filename: Response_to_Reviewers_auresp_2.docx [file pone.0337342.s006.docx]

**Response to Reviewers**

Dear Editors and Reviewers,

Please find below our thorough and thoughtful responses to the invaluable feedback the Editor and Reviewers have provided. Each comment has been carefully considered, and we have taken diligent measures to address every query raised. Our commitment to improving the quality of this manuscript is evident in the revisions we have made.

On behalf of all the authors, I express my sincere gratitude to the Reviewers and the esteemed Editor for their constructive input and valuable suggestions. Their guidance has undoubtedly played a significant role in refining this piece of work. We are genuinely appreciative of this opportunity to enhance our research, and we eagerly look forward to sharing the enhanced and polished version with the esteemed readership of the journal in the near future.

**R: Reviewer	C: Comment(s)**

| **Comments** | **Authors’ response** | **Changes made** | **Reference in Manuscript**  **(Page Numbers)** |
| --- | --- | --- | --- |
| **1. Abstract Revision Notes**  R1C1.1. The research problem (gap) has not been explicitly stated. The abstract directly states that assessment is important, but does not adequately highlight the shortcomings of previous research.  R1C1.2. The research objectives are not explicitly stated.  R1C1.3. The research methods are clear enough, but the technical sections (homogeneity, SD, reliability) are too detailed for the abstract.  R1C1.4. The research results are presented, but they would be better presented concisely without excessive statistical detail.  R1C1.5. The implications/contributions of the research are not clearly stated. | R1C1.1. Thank you for your insightful observation and for sharing your thoughts to improve its quality.  R1C1.2. Thank you for your observation. Changes have been made accordingly.  R1C1.3. Thank you for highlighting this point. It helps us see where improvement is needed.  R1C1.4. Your constructive critique adds significant value. Changes have been made to reflect your suggestions.  R1C1.5. Thank you for your guidance. | R1C1.1. The research gap has been revised in response to the reviewer’s comment.  R1C1.2. The research objectives have been revised to clearly articulate the objectives of the study  R1C1.3. We have revised the abstract by removing excessive technical details to make it more concise and reader-friendly.  R1C1.4. We appreciate this feedback. The results have been condensed to highlight the key findings without unnecessary statistical detail  R1C1.5. Thank you for this observation. We have now stated the implications and contributions of the research to better reflect its significance. | R1C1.1. The revision can be seen in the following page, Page No. 2  R1C1.2. The revision can be seen in the following page, Page No. 2  R1C1.3. The revision can be seen in the following page, Page No.2  R1C1.4. The revision can be seen on the following page, Page No.2  R1C1.5. The revision can be seen in the following page, Page No. 2 |
| **2. Keyword Revision Notes**  R1C2.1. Be concise (3–6 keywords) in accordance with common practice in international journals.  R1C2.2. Avoid long phrases; keywords should be words or short phrases, not descriptive sentences.    R1C2.3. Prioritize frequently used terms in indexing (for easy searchability in databases like Scopus).  keyword suggestions:  Keywords: PROMPTa; Alternative assessment; Peer feedback; Mentor feedback; Expert feedback; Writing skills; ICT. | R1C2.1.Thank you for your relevant observation.  R1C2.2. We appreciate the suggestion. The changes have been addressed.  R1C2.3. Thank you for this helpful recommendation. | R1C2.1. We have reduced the number of keywords in accordance with common practice in international journals.  R1C2.2. We have revised the keywords accordingly. Overly long phrases have been replaced with more concise words  R1C2.3. We have replaced the terms with frequently used and relevant terms to improve indexing and searchability in databases like Scopus | R1C2.1. The revision can be seen in the following page, Page No. 2  R1C2.2. The revision can be seen in the following page, Page No. 2  R1C2.3. The revision can be seen in the following page, Page No. 2 |
| **3. Introduction Revision Notes**  R1C3.1. Too long and Repetitive  R1C3.2. Lack of Systematic Structure  R1C3.3. Too Much Theory at the Beginning  R1C3.4. Insufficiently Integrated References  R1C3.5. Absence of Explicit Statement of Research Gaps | R1C3.1. Thank you for the feedback.  R1C3.2. We appreciate this guidance as it improves the clarity of the study.  R1C3.3. We appreciate this suggestion.  R1C3.4. We acknowledge this concern and are grateful for the observation.  R1C3.5. Thank you for this important note. | R1C3.1. The introduction has been streamlined by removing redundancies and keeping it more focused. shortened and organized for better clarity.  R1C3.2. The introduction is now reorganized to follow a clear, logical flow that guides readers effectively.  R1C3.3. We have reduced the amount of theory.  R1C3.4. Thank you for highlighting this. We have integrated references more effectively to support arguments.  R1C3.5. We have added a clear statement of the research gaps to justify the relevance and contribution of the study. | R1C3.1. The revision can be seen in the following page, Page No. 3- 5  R1C3.2. The revision can be seen in the following page, Page No. 3- 5  R1C3.3. The revision can be seen in the following page, Page No. 3- 5  R1C3.4. The revision can be seen in the following page, Page No. 4  R1C3.5. The revision can be seen in the following page, Page No. 3- 5 |
| **4. Method Revision Notes**  R1C4.1. Poorly organized structure  The methodology mixes method descriptions, pre-test results, and reliability analyses, which should be in the results section, not the methodology.  There are overly long narrative sections (e.g., explaining students' writing challenges) that would be more appropriate in the discussion section.  R1C4.2. Too much operational detail  Mentioning specific intervention dates (e.g., "July 17, 2023 to September 9, 2023") is unnecessary in the methodology unless there is a specific reason.  The weekly phases are too detailed; they should be summarized as the main phases of the intervention.  R1C4.3. Unclear subsections  The methodology should be divided into clear sections: Research Design, Participants, Instruments, Procedures, Data Analysis.  Currently, the methodology does not follow this structure, making it difficult to follow.  R1C4.4. Lack of focus on methodological justification.  There is no explanation of why mixed methods were chosen and how this method enhances the validity of the findings.  There is no justification for selecting the sample size other than "average standard deviation", which needs to be linked to the research justification.  **5. limitations and implications Revision Notes**  R1C5.1. No clear statement of limitations.  The section begins with the sentence "This study acknowledges several limitations," but then jumps to the conclusion without fully addressing the limitations of the study.  R1C5.2. Mixed sections.  The text includes conclusions and a rehash of the results (e.g., "This study highlights the effectiveness of..."), when it should only outline the limitations of the study and implications for future research or educational practice.  R1C5.3. Lack of focus on implications.  The implications are still a general narrative about the effectiveness of PROMPTa, rather than clear recommendations for educators, policymakers, or future research.  R1C5.4. Lack of conciseness.  There is a lot of repetition that should be condensed.  **6. Conclusion Revision Notes**  R1C6.1. Too long and repetitive  Many concepts are repeated, for example, the effectiveness of PROMPTa is mentioned repeatedly.  R1C6.2. Including new information that should be in "Limitations and Implications"  The section on "infrastructure limitations, accessibility, and digital literacy" is more appropriately placed in the limitations/implications section, not the conclusion.  R1C6.3. Lack of focus  The conclusion should reiterate the main findings, research contributions, and brief recommendations, not rehash the methodology or lengthy discussion.  R1C6.4. Lack of academic punch line  There is no clear concluding sentence about the scientific contributions and future research directions. | R1C4.1. Thank you for the keen observation.  R1C4.2. We appreciate this suggestion.  R1C4.3. Thank you for pointing this out.  R1C4.4. Thank you for your helpful suggestion for the enhancement of the quality of the paper.  R1C5.1. Thank you for highlighting this. It is highly appreciated.  R1C5.2. We appreciate the suggestion.  R1C5.3. Thank you for pointing out this need for clarification.  R1C5.4. Thank you. It is acknowledged.  R1C6.1. Thank you for the feedback.  R1C6.2. We appreciate the suggestion.    R1C6.3. Thank you for the valuable insight.  R1C6.4. Thank you for the suggestion. | R1C4.1.We have restructured the methodology by separating methodological details from results and moving contextual narratives to the discussion section for better clarity  R1C4.2. We appreciate the feedback. We have removed unnecessary specifics as pointed out and condensed weekly details into broader intervention phases to keep the methodology concise.  R1C4.3. The revised methodology now follows a clear structure.  R1C4.4. Thank you for pointing this out. We have provided a clear rationale for adopting mixed methods and justified the sample size in relation to research objectives and the validity of findings.  R1C5.1 We have revised the section to explicitly state the study’s limitations before moving to the conclusion.  R1C5.2. We have separated limitations from conclusions and results to ensure each section serves its proper purpose.  R1C5.3. The implications have been revised to provide clear, actionable recommendations for educators, policymakers, and future researchers  R1C5.4. We have revised the section to make it more concise by removing unnecessary repetition and keeping the focus sharp.  R1C6.1. Thank you for the observation. We have shortened the conclusion by removing repetitive points, especially the repeated mention of PROMPTa’s effectiveness, to keep it more focused  R1C6.2. I have moved the discussion on infrastructure, accessibility, and digital literacy to the limitations/implications section, where it fits better.  R1C6.3. We have revised the conclusion to highlight only the main findings, key contributions, and brief recommendations, avoiding unnecessary methodological or discussion details.  R1C6.4. The revised version has strengthened the conclusion by adding a clear final sentence that highlights the study’s contributions and points toward future research directions | R1C4.1. The revision can be seen in the following page, Page No. 7  R1C4.2. The revision can be seen in the following page, Page No. 15  R1C4.3. The revision can be seen in the following pages, Page No. 12-23  R1C4.4. The revision can be seen in the following pages Page No. 12-13  R1C5.1 The revision can be seen in the following pages, Page No. 26 and 27  R1C5.2. The revision can be seen in the following page, Page No. 27  R1C5.3. The revision can be seen in the following page, Page No. 28  R1C5.4. The revision can be seen in the following pages, Page No. 26 and 27  R1C6.1. The revision can be seen in the following page, Page No. 29  R1C6.2. The revision can be seen in the following page, Page No. 29  R1C6.3. The revision can be seen in the following pages, Page No. 29-30  R1C6.4. The revision can be seen in the following page, Page No. 30 |
| R2C1. Theoretical framework is now well-integrated, combining SCT, Constructivism, and Constructionism into a cohesive model.  R2C2. The methodology is clearly explained, including intervention phases and the role of PROMPTa.  R2C3. Quantitative results are robust, and qualitative analysis provides rich contextual insights.  R2C4. Practical relevance is high, especially for low-resource educational settings.  R2C5. Inclusion of anonymized feedback samples offers transparency into the assessment process.  Suggestions for further improvement:  R2S1. Consider explicitly reporting effect sizes for key statistical comparisons to complement p-values.  R2S2. In the Discussion, strengthen the explicit connection between findings and how they advance or challenge prior literature.  R2S3. Briefly address scalability and feasibility of PROMPTa in varied institutional contexts, especially where ICT infrastructure is limited.  R2S4. Split a few longer paragraphs in the Introduction and Literature Review for improved readability | R2C1. We sincerely appreciate your suggestions that helped to strengthen our paper.  R2C2. Thank you for this acknowledgement.  R2C3. We thank you for acknowledging the contextual grounding of the study.  R2C4. Your keen observation is deeply appreciated.  R2C5. Thank you for your detailed observations.  R2S1. Your comments reflect both care and expertise.  R2S2. Thank you for pointing this out. It points the work in the right direction for further refinement  R2S3. Thank you for this point.  R2S4. Thank you for highlighting this. | R2C1. (NIL)  R2C2. (NIL)  R2C3. (NIL)  R2C4. (NIL)  R2C5. (NIL)  R2S1. As per the suggestion, we have reported the effect sizes alongside p-values to provide a fuller understanding of the statistical comparisons  R2S2. As per the suggestions, we have strengthened the discussion by explicitly linking the findings to how they support, extend, or challenge previous studies.  R2S3. We have briefly addressed the scalability of PROMPTa and its feasibility in contexts with limited ICT infrastructure  R2S4.We have split longer paragraphs in these sections to enhance readability and flow | R2C1. (NIL)  R2C2. (NIL)  R2C3. (NIL)  R2C4. (NIL)  R2C5. (NIL)  R2S1. The revision can be seen in the following page, Page No. 21  R2S2. The revision can be seen in the following pages,  Page No. 26.  R2S3. The revision can be seen in the following pages, Page No. 28  R2S4. The revision is evident in the mentioned sections. |
