# Supplementary material for: PROMPTa—A multifaceted assessment approach to develop writing skills
Source: PLoS One. 2025 Dec 2;20(12):e0337342. doi: 10.1371/journal.pone.0337342 (PMC12671783; doi:10.1371/journal.pone.0337342)
Supplement: Attachment — Submitted filename: Response_to_Reviewers_auresp_4.docx [file pone.0337342.s009.docx]

**Response to Reviewers**

Dear Reviewer,

We thank you for your constructive feedback on our manuscript. We greatly appreciate the time and the effort taken to highlight areas requiring revision. We have carefully considered each of your suggestion and made revision accordingly. Thank you for your invaluable inputs in making this manuscript ready for publication as per the International standards.

| **Comments** | **Authors’ response** | **Changes made** | **Reference in Manuscript**  **(Page Numbers)** |
| --- | --- | --- | --- |
| 1. Include more details on how PROMPTa ensures inter-rater fairness and consistency in real classroom setting. | We thank the reviewer for this insightful suggestion. The revised section now elaborates on how PROMPTa ensures inter-rater fairness and consistency in real classroom contexts. | To ensure fairness and consistency in scoring, PROMPTa followed several accuracy checks. In this context, there were three levels of assessors: peers, mentors, and experts. Peers received orientation and practice using sample writings and clear explanations of the rubrics created by the researcher. The mentor, who was also the researcher, applied the same validated rubrics when providing scoring and feedback. Experts, who had validated the rubrics, also evaluated the final drafts to ensure consistency across all stages. While scoring PROMPTa offered a structured digital platform where all assessors could refer to the same rubrics. Student identities remained anonymous to prevent bias, and the platform encouraged specific, evidence-based feedback instead of subjective opinions. | The revision can be seen in the following page, Page No. 13 |
| 2. Clarify whether the tool’s usability and accessibility were measured quantitatively (e.g., user satisfaction scales) | We appreciate the reviewer’s helpful observation. The revision clarifies that while standardised user-satisfaction scales were not employed, usability and accessibility were examined qualitatively through open–ended questionnaires and interviews. | Usability and accessibility were examined qualitatively through open-ended questionnaires and interviews focusing on navigation, ease of use, and accessibility of submission, feedback, and anonymity features. Though no standardized user-satisfaction scales were employed, post-intervention responses indicated strong usability: 82% found the prompts engaging and 88% appreciated the platform’s accessibility. These, supported by qualitative feedback show that PROMPTa was both user-friendly and accessible. | R1C1.1. The revision can be seen in the following page, Page No. 13 |
| 3.  A brief reflection on scalability or adaptability to other educational contexts could strengthen the implications section. | We thank the reviewer for this valuable recommendation. A concluding point has been added to reflect PROMPTa’s scalability and adaptability. | **Scalability and Adaptability:** PROMPTa demonstrates strong potential for adaptation beyond its original setting. Its rubric based, flexible design allows customization across subjects, grade levels, and institution types. | The revision can be seen in the following page, Page No. 27 |
